# Impact of Co-Reactants in Atomic Layer Deposition of High-κ Dielectrics on Monolayer Molybdenum Disulfide

**DOI:** 10.1021/acsanm.5c00901

**Published:** 2025-04-01

**Authors:** Brendan F. M. Healy, Sophie L. Pain, Marc Walker, Nicholas E. Grant, John D. Murphy

**Affiliations:** † School of Engineering, University of Warwick, Coventry CV4 7AL, U.K.; ‡ Department of Physics, University of Warwick, Coventry CV4 7AL, U.K.

**Keywords:** monolayer MoS_2_, transition metal dichalcogenides, ALD, high-κ dielectrics, ozone, O_2_ plasma

## Abstract

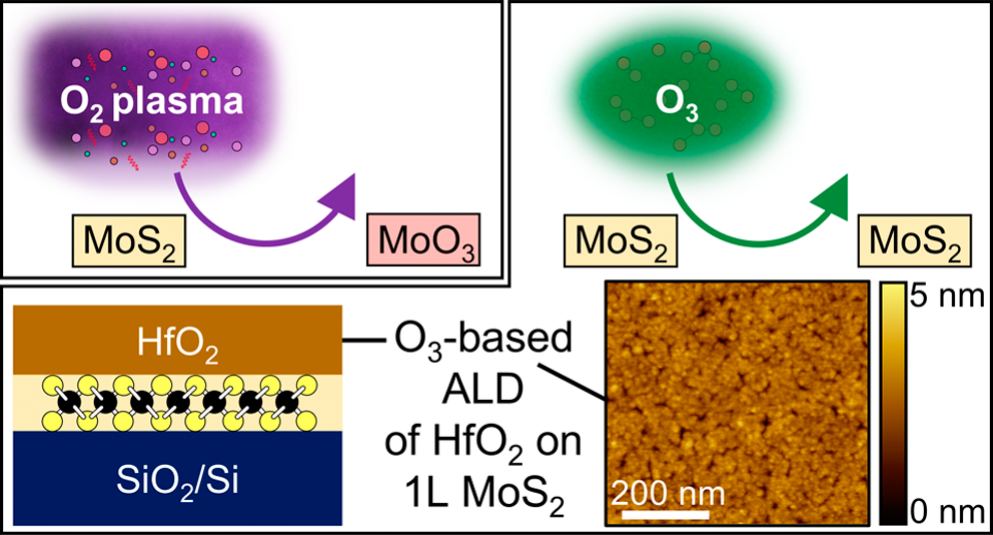

The integration of single-layer transition metal dichalcogenides (TMDCs) in nanoscale field-effect transistor devices requires the deposition of a high dielectric constant (high-κ) material to act as the gate dielectric. Traditional thermal atomic layer deposition (ALD) is commonly used to deposit dielectrics on three-dimensional substrates, but ALD of high-κ materials on monolayer TMDCs is more challenging. Thermal ALD with water (H_2_O) co-reactant often results in incomplete and nonuniform dielectric growth on atomically thin TMDCs, owing to a chemically inert basal plane. The development of alternative ALD processes for the realization of dielectric layers on monolayer TMDCs is therefore important. Here, we study oxygen (O_2_) plasma and ozone (O_3_) as co-reactants for the ALD of aluminum oxide (Al_2_O_3_) and hafnium dioxide (HfO_2_) on monolayer molybdenum disulfide (1L MoS_2_) films. By employing a robust characterization process that combines atomic force microscopy, Raman/photoluminescence spectroscopy, and X-ray photoelectron spectroscopy, we reveal growth of high-κ dielectrics by plasma-enhanced ALD with O_2_ plasma oxidant damages the underlying 1L MoS_2_ via oxidation to molybdenum trioxide (MoO_3_). No significant deleterious oxidation to MoO_3_ is observed following O_3_-based deposition on 1L MoS_2_, and we demonstrate the growth of HfO_2_ via thermal ALD with O_3_ co-reactant. This work reveals the impact of ALD processes on 1L MoS_2_ during the growth of high-κ dielectrics, highlighting O_3_-based thermal ALD as a potential route for the integration of dielectric layers on 1L MoS_2_ for nanoscale optoelectronic device fabrication.

## 1 Introduction

Ever since the initial isolation of graphene, two-dimensional (2D) materials have been widely studied. The lack of an intrinsic bandgap in graphene limits its suitability for nanoscale optoelectronic technologies. A number of transition metal dichalcogenides (TMDCs), a class of layered materials, possess an intrinsic bandgap and have therefore attracted significant research interest as novel 2D semiconducting alternatives to graphene. TMDCs possess the chemical formula MX_2_, where M is a transition metal atom and X is a chalcogen atom. Molybdenum disulfide (MoS_2_) is a representative semiconducting TMDC that exhibits promising chemical and physical behavior at single-layer thickness. In bulk form, MoS_2_ possesses an indirect bandgap of ∼1.2 eV, whereas monolayer MoS_2_ (1L MoS_2_) has a direct bandgap of ∼1.8 eV.^1^ As such, 1L MoS_2_ is a promising candidate for a range of nanoscale optoelectronic applications, including photodetection devices,^2^ photovoltaic cells,^3^ and field-effect transistors (FETs).^4,5^ The FET architecture is ubiquitous in modern electronics, and is a crucial component in integrated circuits. With a direct bandgap, exceptional current on/off ratio, and superior charge carrier mobility compared to the bulk structure, 1L MoS_2_ is a desirable channel material for fast-switching FETs. The generation of a MoS_2_-based FET often requires deposition of a high dielectric constant (high-κ) material, often aluminum oxide (Al_2_O_3_) or hafnium dioxide (HfO_2_), to function as the gate dielectric and provide electrostatic control over the conductivity in the MoS_2_ channel. Optimal nanoscale device performance requires thin, continuous, and uniform dielectric layers with minimal deleterious impact on the underlying 1L MoS_2_, as well as a high-quality dielectric/MoS_2_ interface.

Thin dielectric films for nanoscale device applications are commonly grown via atomic layer deposition (ALD), a variant of chemical vapor deposition (CVD) that offers precise control over the deposited film thickness. In a typical ALD process, gaseous precursor and co-reactant molecules are alternately pulsed onto the substrate. After each pulse, purge steps remove excess precursor/co-reactant and any reaction byproducts, ensuring the self-limiting chemical reaction of the two reactant species on the substrate surface to yield the desired dielectric film.^6^ Direct ALD, where no pretreatment of the surface is performed before deposition, can achieve high-quality, highly uniform dielectric layers on standard three-dimensional (3D) substrates, as dangling bonds on the surface facilitate initial chemisorption of the precursor molecules. However, the direct ALD of dielectrics on monolayer TMDCs is difficult, owing to an absence of out-of-plane dangling bonds on the chemically inert basal plane. Therefore, with insufficient reaction sites to facilitate chemisorption of ALD reactants, direct ALD on single-layer TMDCs is dominated by a physisorption mechanism, where the ALD precursor and co-reactant physically attach to the monolayer surface via a weak intermolecular force. Hence, ALD-dielectric films grown directly on 1L MoS_2_ at standard operating temperatures (∼150–250 °C) are typically incomplete and discontinuous, particularly at sub-10 nm thicknesses.^7,8^ A range of predeposition surface functionalization techniques have been shown to enhance the growth of high-κ dielectrics on MoS_2_, including plasma pretreatment,^7,9^ ultraviolet-ozone (UV–O_3_) exposure^10,11^ and seeding layers.^12^ Despite improved growth of ALD-dielectrics, such pretreatments often introduce defects in the underlying MoS_2_, so an optimized direct ALD process remains desirable.

The growth of high-κ dielectric films on MoS_2_ is sensitive to the ALD process conditions. As well as the reactant pulse times, deposition temperature, and reaction pressure, the choice of co-reactant molecule will influence the nucleation and evolution of the high-κ film.^13^ Thermal ALD using water (H_2_O) vapor as the co-reactant is the most widely studied ALD procedure for the direct growth of dielectric materials on 1L MoS_2_, but often yields nonuniform film morphologies. We have previously reported the growth of Al_2_O_3_ and HfO_2_ films on 1L MoS_2_ via H_2_O-based thermal ALD at 200 °C, revealing incomplete surface coverage of both dielectrics after 200 ALD cycles.^14^ Ozone (O_3_) has been proposed as an alternative oxidant.^15^ Compared to ALD via H_2_O, O_3_-based thermal ALD of Al_2_O_3_ on conventional silicon (Si) substrates is known to offer greater reactivity and a lower density of defects in the resultant film, owing to the superior oxidative power of O_3_.^15^ It is hoped that similar benefits may be experienced in the ALD of Al_2_O_3_ on MoS_2_,^16,17^ yet deposition of dielectrics on TMDCs from an O_3_ co-reactant is poorly researched. Moreover, despite numerous reports regarding UV–O_3_ treatment of MoS_2_,^10,11,18^ the impact of O_3_ on 1L MoS_2_ during an ALD process has not been well established.

The realization of high-quality dielectric films on standard 3D substrates has also been enabled by the advent of plasma-enhanced ALD (PEALD).^19^ Traditional thermal ALD involves a vapor phase co-reactant such as H_2_O or O_3_ and relies solely on thermal energy to drive the chemical reactions. Conversely, PEALD utilizes plasma activation, and a plasma is employed as the co-reactant. During each PEALD cycle, the substrate surface is exposed to highly reactive radical species generated in the plasma. Common plasmas include those produced from oxygen (O_2_), nitrogen (N_2_), and hydrogen (H_2_). The high reactivity of the plasma species enables lower processing temperatures than those typically required by classical ALD systems.^20^ Current research into the use of PEALD for the growth of dielectric films on TMDC surfaces is limited.^21−23^ Price et al. reported PEALD of uniform Al_2_O_3_ and HfO_2_ films on exfoliated multilayer MoS_2_ via O_2_ plasma at relatively modest temperatures (between 120 and 320 °C).^21^ However, the authors observed plasma-induced damage to the MoS_2_ surface when depositing HfO_2_ via PEALD on CVD-grown 1L MoS_2_, despite employing a remote plasma delivery.^22^ Further research attention is therefore required to understand how PEALD of dielectrics impacts single-layer TMDC materials so that any parasitic effects of the plasma exposure process can be mitigated and the benefits of PEALD maximized.

In this work, we study alternative co-reactants for the direct ALD of high-κ dielectrics on 1L MoS_2_. We present a comprehensive comparison of the direct ALD of Al_2_O_3_ and HfO_2_ on CVD-1L MoS_2_ via O_2_ plasma and O_3_ oxidizing agents. We combine Raman and photoluminescence (PL) measurements, atomic force microscopy (AFM), and X-ray photoelectron spectroscopy (XPS) to assess how each deposition process impacts the structural and optical properties of both the dielectric film and the 1L MoS_2_.

## 2 Experimental Details

### 2.1 Chemicals and Materials

A 1 × 1 cm^2^ hexagonal phase undoped 1L MoS_2_ film (>99%) grown on silicon dioxide/silicon (SiO_2_/Si) via atmospheric pressure chemical vapor deposition (APCVD) was obtained from 2D Semiconductors. This 1L MoS_2_ film, received in vacuum-sealed packaging, was cleaved into at least nine smaller samples of approximately equal size (∼0.3 × 0.3 cm^2^) and the cleaved samples were stored in the dark in a vacuum desiccator to minimize exposure to the ambient environment. The monolayer nature of the MoS_2_ film was verified via AFM imaging and Raman mapping measurements, as presented in Section S1 of the Supporting Information.

### 2.2 High-κ Dielectric Deposition

ALD of Al_2_O_3_ and HfO_2_ was conducted in a Veeco Fiji G2 system with plasma-enhanced and thermal capabilities. All depositions were performed at 200 °C and argon was employed as the carrier and purging gas. In each process, 200 ALD cycles and two different co-reactants were investigated—O_2_ plasma and O_3_. A high-density 13.56 MHz radio frequency (RF) O_2_ plasma is generated via a remote inductively coupled plasma (ICP) source at a power of 300 W. Al_2_O_3_ was deposited using a trimethylaluminum (TMA) precursor, held at room temperature. Tetrakis(dimethylamido)hafnium (TDMAH) precursor, heated to 75 °C, was used for the growth of HfO_2_. O_3_ is generated prior to deposition from flowing O_2_ via a generator within the ALD system. For all depositions of Al_2_O_3_ and HfO_2_, the precursor delivery lines were heated to 150 °C. Specific deposition parameters are detailed in Table 1. To investigate the impact of the oxidant, two 1L MoS_2_ samples were each subjected to 200 cycles of a modified ALD process that omitted the introduction of a metal precursor—one with only pulses of O_2_ plasma exposure and one comprising only pulses of O_3_.

**Table 1:** Summary of the Parameters Associated with Each of the ALD Processes Studied Here

parameter	PEALD of Al_2_O_3_	O_3_-ALD of Al_2_O_3_	PEALD of HfO_2_	O_3_-ALD of HfO_2_	O_2_ plasma exposure	O_3_ exposure
precursor pulse duration (s)	0.06	0.06	0.25	0.25		
precursor purge duration (s)	4	10	5	15		
co-reactant pulse duration (s)	6	0.15	6	0.15	6	0.15
co-reactant intensity (W)	300		300		300	
co-reactant purge duration (s)	4	10	5	15	4	10

### 2.3 Atomic Force Microscopy

A Bruker Dimension Icon in the PeakForce Tapping Mode with a ScanAsystAir tip (nominal tip length: 115 μm, tip radius: 2 nm and spring constant: 0.4 N m^–1^) was used to image surface topographies.^24^ All AFM images were acquired with 256 lines per scan at a scan rate of 0.5 Hz to yield a suitable resolution. Processing and analysis of the AFM images was performed in the Gwyddion 2.60 software package.^25^ The Fiji distribution of the ImageJ software package was used to estimate the surface coverage of the ALD-dielectric films by converting the AFM images to 8 bit grayscale and then binary images via the software’s thresholding algorithm.^26^

### 2.4 Raman and PL Spectroscopy

All Raman and PL data were obtained at room temperature via a Renishaw inVia Reflex Raman microscope in standard confocal mode with a 532 nm excitation laser at 0.1% of maximum power (∼0.18 μW), with these parameters established as the optimum laser exposure conditions for 1L MoS_2_ in a previous study.^27^ A 50× Leica objective lens was used, with a numerical aperture of 0.75 and a grating with 1800 lines/mm. This optical configuration was also used to capture optical micrographs of the samples. Single-spot Raman data were the sum of 4 accumulations of 5 s each, with the reported associated PL data being the sum of 4 accumulations of 10 s each. Multipeak Lorentzian fitting of single-spot PL spectra was performed, with the exciton and trion peak energies constrained within approximate initial estimates and line widths allowed to vary freely. Where Raman spectra were seen to exhibit characteristic MoS_2_ E^1^_2g_ and A_1g_ features, the signal was fitted with a superposition of two Lorentzian functions. For samples where no explicit MoS_2_ Raman peaks were detected, raw spectra were plotted without any fitting. Raman and PL mapping data were also obtained for each sample, collected over an area of 12 × 12 μm^2^ with a step size of 0.5 μm. The PL spectrum at each point in the map was fitted with a single Lorentzian curve. Similarly, a superposition of two Lorentzian curves was fitted to each Raman spectrum to encompass the two signature MoS_2_ peaks. The exposure conditions used to acquire PL/Raman data following each ALD process were identical to those used to measure the corresponding untreated MoS_2_ sample. All data were captured via the Renishaw WiRE 3.1 software package, and any cosmic-ray features were removed where necessary.

### 2.5 X-ray Photoelectron Spectroscopy (XPS)

XPS was conducted at the Photoemission Research Technology Platform at the University of Warwick in a Kratos Axis Ultra delay-line detector (DLD) spectrometer. Samples were mounted via electrically conductive carbon (C) tape on to a nonmagnetic, stainless-steel bar. A monochromated Al Kα X-ray (1.487 keV) source was used, with measurements performed at room temperature at a takeoff angle of 90° with respect to the sample surface. A charge neutralizer was used to alleviate any charging effects and all XPS data were charge referenced to the C 1s core level of adventitious C contamination at 284.8 eV. Core level spectra were collected from an analysis area of 300 × 700 μm^2^ with a pass energy of 20 eV and the resolution of the detector was 0.4 eV. Fitting procedures to extract peak positions and relative atomic concentrations were performed in the CasaXPS software package, supported by the NIST XPS database.^28^ Core level spectra were fitted with a relevant background, with Shirley, linear, and Tougaard backgrounds used where appropriate. Where we present XPS core level spectra, the associated figure caption indicates the relevant background that was used in the fitting procedure. Mixed Gaussian–Lorentzian (Voigt) line shapes were primarily used, with a finite, asymmetric Lorentzian line shape used to fit any Mo 3d doublets. The core level peak areas were corrected using the Scofield relative sensitivity factors, considering the photoelectron mean free paths and photoionization cross sections of the core levels.

## 3 Results and Discussion

We investigate the growth of Al_2_O_3_ and HfO_2_ on CVD-1L MoS_2_ via PEALD, where O_2_ plasma is used in place of a traditional oxidant, and via thermal ALD with O_3_ co-reactant. To isolate the effects of exposure to O_2_ plasma or O_3_ that may occur during ALD, we also subjected separate 1L MoS_2_ samples to modified ALD recipes that omitted the introduction of any metal precursor: one process comprised solely O_3_ pulses and a second recipe pulsed O_2_ plasma only. All ALD processes were performed at 200 °C with the same number of cycles (200).

### 3.1 Structural Characterization

**Figure 1:**
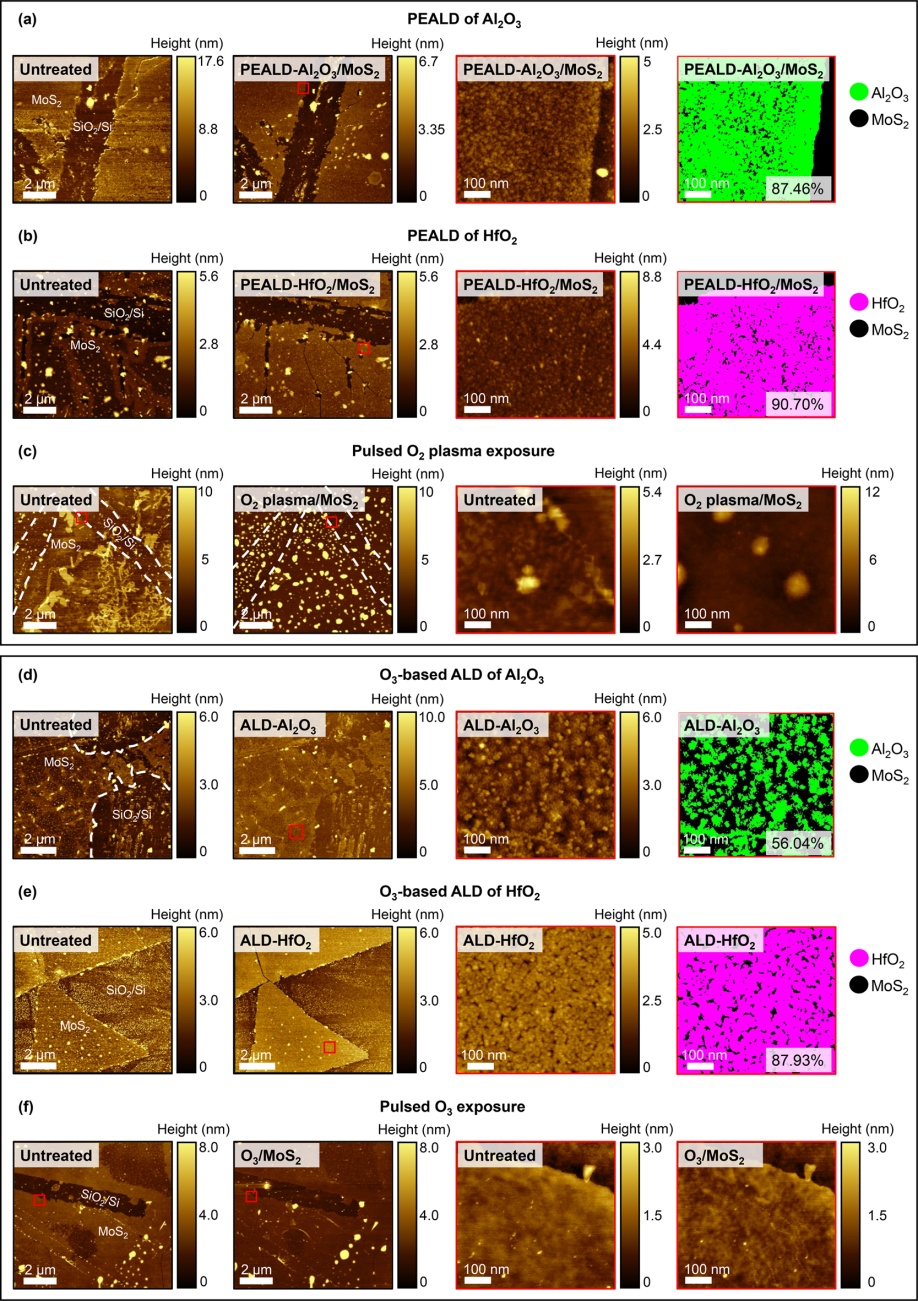
Low- and high-magnification AFM images for PEALD of (a) Al_2_O_3_ and (b) HfO_2_, (c) pulsed O_2_ plasma exposure, O_3_-based ALD of (d) Al_2_O_3_ and (e) HfO_2_, and (f) pulsed O_3_ exposure. Red squares highlight the areas from which high-magnification images were acquired. For deposition of Al_2_O_3_ or HfO_2_ (a,b,d,e), estimated surface coverage maps are displayed.

The morphologies of the same regions of each sample were imaged via AFM at various stages of processing. Low-magnification topographic AFM images acquired from each 1L MoS_2_ sample before and after each ALD process are shown in Figure 1. For samples where Al_2_O_3_ or HfO_2_ was deposited, high-magnification AFM images and maps of the estimated coverage of the dielectric film are also presented. For pulsed O_2_ plasma and O_3_ treatments, high-magnification images before and after treatment are shown. The presence of bright spots and features in the AFM images of the untreated 1L MoS_2_ samples indicates varying degrees of surface contamination that exist prior to any ALD treatment, possibly unreacted material from the CVD synthesis or adsorbed impurities.

Exposure to O_2_ plasma can impact MoS_2_ in two primary ways: chemical modification of the MoS_2_ lattice or physical etching of MoS_2_ layers.^29,30^ Physical etching usually occurs when highly energetic species in the plasma impact directly on the MoS_2_. In the PEALD configuration used here, the 1L MoS_2_ sample is held on a heated chuck ∼35 cm from the remote plasma source. As the plasma diffuses over the considerable distance from the source to the 1L MoS_2_ sample, the most damaging species in the plasma, typically high-energy electrons and ions, are likely to recombine via charge exchanges and recombination reactions with reactive surfaces, namely the walls of the reactor.^31^ As a result, no appreciable physical etching of the 1L MoS_2_ is expected from exposure to a remotely generated plasma.^32^ Following PEALD of Al_2_O_3_ and HfO_2_, no significant morphological changes to the 1L MoS_2_ samples were observed in the low-magnification AFM images (second column in Figure 1a,b), confirming no observable physical etching at the scale of the magnification occurs during PEALD of either dielectric. The granular morphology observed in the high-magnification AFM images (third column in Figure 1a,b) suggests the PEALD-Al_2_O_3_ and PEALD-HfO_2_ films have developed via an island growth mode, with incomplete dielectric coalescence yielding ∼90% surface coverage in each case. The surface roughness of gate dielectric layers is important for the performance of nanoscale MoS_2_-based FET devices, with increased roughness reducing the carrier mobility and impeding device performance.^33^ The root-mean-square (RMS) surface roughness of the PEALD-Al_2_O_3_ film was ∼0.4 nm, with the PEALD-HfO_2_ having an RMS roughness of ∼0.6 nm. The RMS roughness and surface coverage values associated with each ALD treatment explored in this study are presented in Table 2, and AFM images showing the areas of each sample used for RMS roughness analysis are displayed in Figure S3 in the Supporting Information. XPS core level spectra provided in Figure S4 in the Supporting Information verify the formation of Al_2_O_3_ and HfO_2_ films. Analysis of the core level spectra reveals the PEALD-Al_2_O_3_ film to be oxygen-deficient, with a stoichiometry of Al_2_O_*x*_ where *x* ∼ 2.4, whereas we estimate the PEALD-HfO_2_ film to be oxygen-rich HfO_*y*_ with *y* ∼ 2.7. The topographic AFM image acquired from the 1L MoS_2_ sample subjected to pulsed O_2_ plasma (second column in Figure 1c) shows the physical structure of the monolayer survives the treatment, with a step height between the monolayer and SiO_2_/Si substrate preserved (Figure S5 in the Supporting Information). The magnitude of this step height does, however, increase from the typical thickness of 1L MoS_2_ (∼0.7 nm) to ∼1.3 nm, which may be indicative of the formation of some molybdenum oxide (MoO_*x*_).^34^ The RMS surface roughness also increases from ∼0.3 nm to ∼5.5 nm due to the emergence of new ∼10 nm-high spots (third and fourth columns in Figure 1c). The dimensions of these surface features are consistent with those of oxide nanoparticles observed on synthetic MoS_2_ following O_2_ plasma treatment.^35^

**Table 2:** Summary of the RMS Roughness and Surface Coverage Values Associated with Each of the ALD Processes Studied Here

treatment	RMS roughness of untreated 1L MoS_2_ (nm)	RMS roughness of treated 1L MoS_2_ (nm)	RMS roughness of dielectric (nm)	surface coverage of dielectric film (%)
PEALD of Al_2_O_3_	0.19		0.42	87.46
PEALD of HfO_2_	0.19		0.57	90.70
pulsed O_2_ plasma	0.26	5.51		
O_3_-based ALD of Al_2_O_3_	0.19		0.70	56.04
O_3_-based ALD of HfO_2_	0.26		0.43	87.93
pulsed O_3_	0.21	0.22		

As with the PEALD processes, AFM imaging indicates no observable physical etching of 1L MoS_2_ at the scale of the magnification occurs during the O_3_-based ALD processes applied here (Figure 1d,e), with the physical structure of the 1L MoS_2_ film maintained in each case. Line profile information presented in Figure S5 in the Supporting Information confirms the 1L MoS_2_ film thickness remains at ∼0.7 nm after pulsed O_3_ treatment. Unlike in the O_2_ plasma-treated sample, no new surface features are present on 1L MoS_2_ after pulsed exposure to O_3_ (Figure 1f), and the RMS roughness of the 1L MoS_2_ surface (∼0.2 nm) is unchanged by the treatment. We find the ALD-Al_2_O_3_ film grown from O_3_ co-reactant has an RMS surface roughness of ∼0.7 nm and covers ∼56% of the MoS_2_ surface, considerably less than the ∼85% surface coverage we have previously reported for ALD-Al_2_O_3_ films deposited on CVD-1L MoS_2_ via thermal ALD with H_2_O oxidant using the same system.^14^ From the core level XPS data provided in Figure S4 in the Supporting Information, we determine the Al_2_O_3_ film grown from O_3_-based ALD to be oxygen-deficient Al_2_O_*x*_ where *x* ∼ 2.5. An oxygen-rich ALD-HfO_*y*_ film (*y* ∼ 2.6 as evaluated from the XPS spectra in Figure S4) was detected in the AFM topographical image following deposition of HfO_2_ from O_3_ co-reactant (third column in Figure 1e), comprising an island morphology and covering ∼90% of the underlying MoS_2_ surface. Despite the island growth, the high degree of coalescence renders the ALD-HfO_2_ film relatively smooth, with an RMS roughness of ∼0.4 nm. The instability of gaseous O_3_ at the 200 °C deposition temperature used here was considered as an explanation for the poorer coalescence of the ALD-Al_2_O_3_ film grown via O_3_. Henning et al. suggested that O_3_ is unstable when used as a co-reactant in ALD reactions at temperatures >100 °C, due to spontaneous decomposition into O_2_ and oxygen radicals (O*).^15,17^ However, Cheng et al. proposed that dissociated O* can be weakly adsorbed on MoS_2_ and provide the requisite reactive sites for ALD of dielectric layers, reporting growth of uniform Al_2_O_3_ following ALD of 30 cycles via O_3_ co-reactant at 200 °C.^16^ Since we achieved ∼90% dielectric surface coverage by O_3_-based ALD of HfO_2_ at 200 °C, we infer that the ALD temperature is not responsible for the poorer surface coverage of the ALD-Al_2_O_3_ film. Instead, differences in the precursor/MoS_2_ surface interaction are likely responsible for the less favorable growth. The adsorption of the bulkier TDMAH precursor on 1L MoS_2_ may be more energetically favorable than that of TMA.^36^

With no appreciable etching apparent in the AFM images, we suspect that any changes to the properties of 1L MoS_2_ samples observed following PEALD or O_3_-based ALD of Al_2_O_3_ or HfO_2_, or indeed pulsed O_2_ plasma or O_3_ exposure, result from oxidation of MoS_2_ to some MoO_*x*_. A more detailed understanding of the growth of high-κ materials on 1L MoS_2_ via PEALD and O_3_-based ALD is developed in our subsequent discussion of the influence of such ALD processes on the optical behavior of 1L MoS_2_.

### 3.2 Optical Properties of Dielectric-Encapsulated 1L MoS_2_

**Figure 2:**
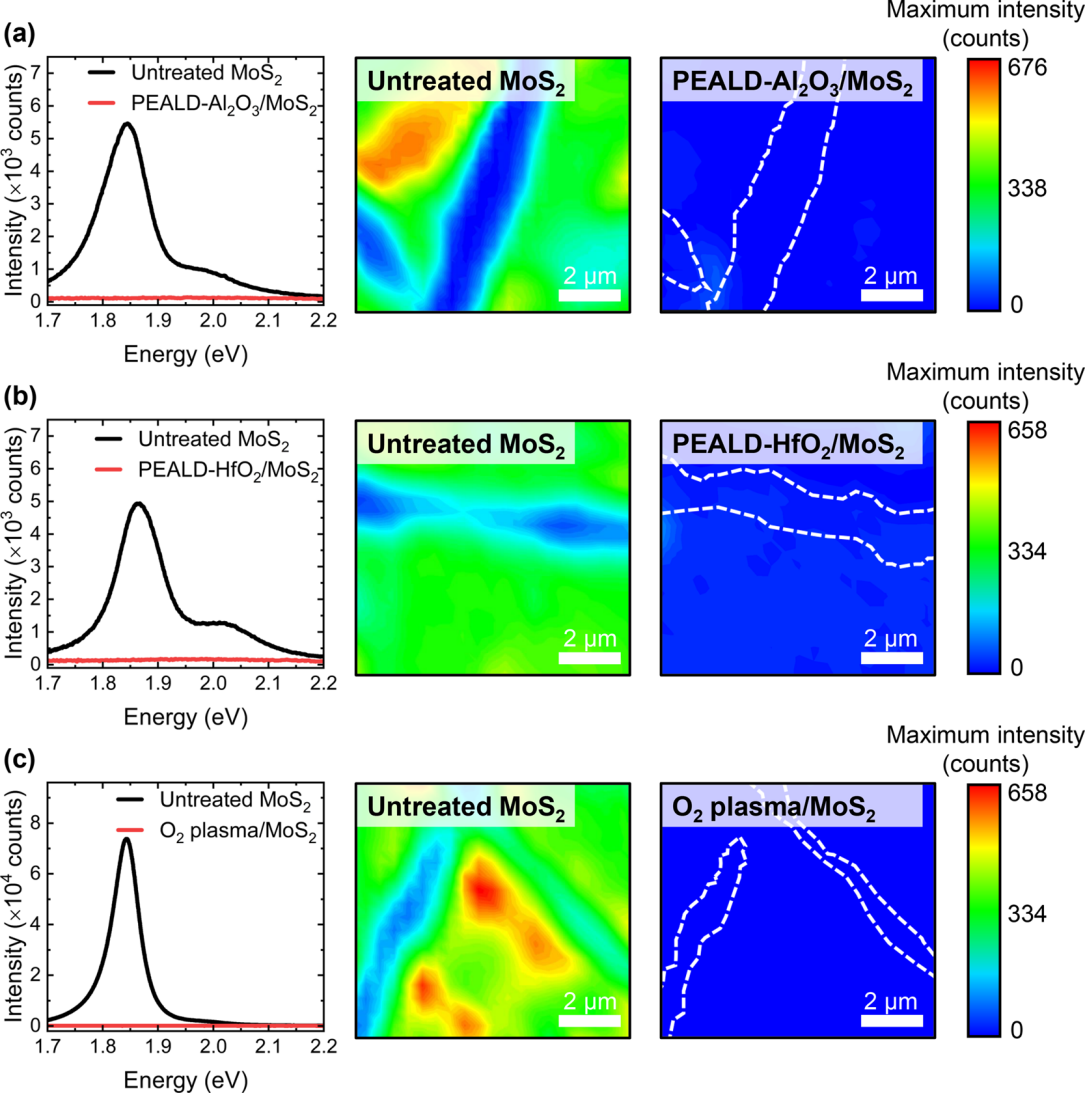
Single-spot PL spectra and maps of the absolute PL maximum intensity before (middle) and after (right) (a) PEALD of Al_2_O_3_, (b) PEALD of HfO_2_, and (c) pulsed O_2_ plasma exposure. The white dashed lines in the PL maps measured after each PEALD treatment indicate the MoS_2_ structure.

**Figure 3:**
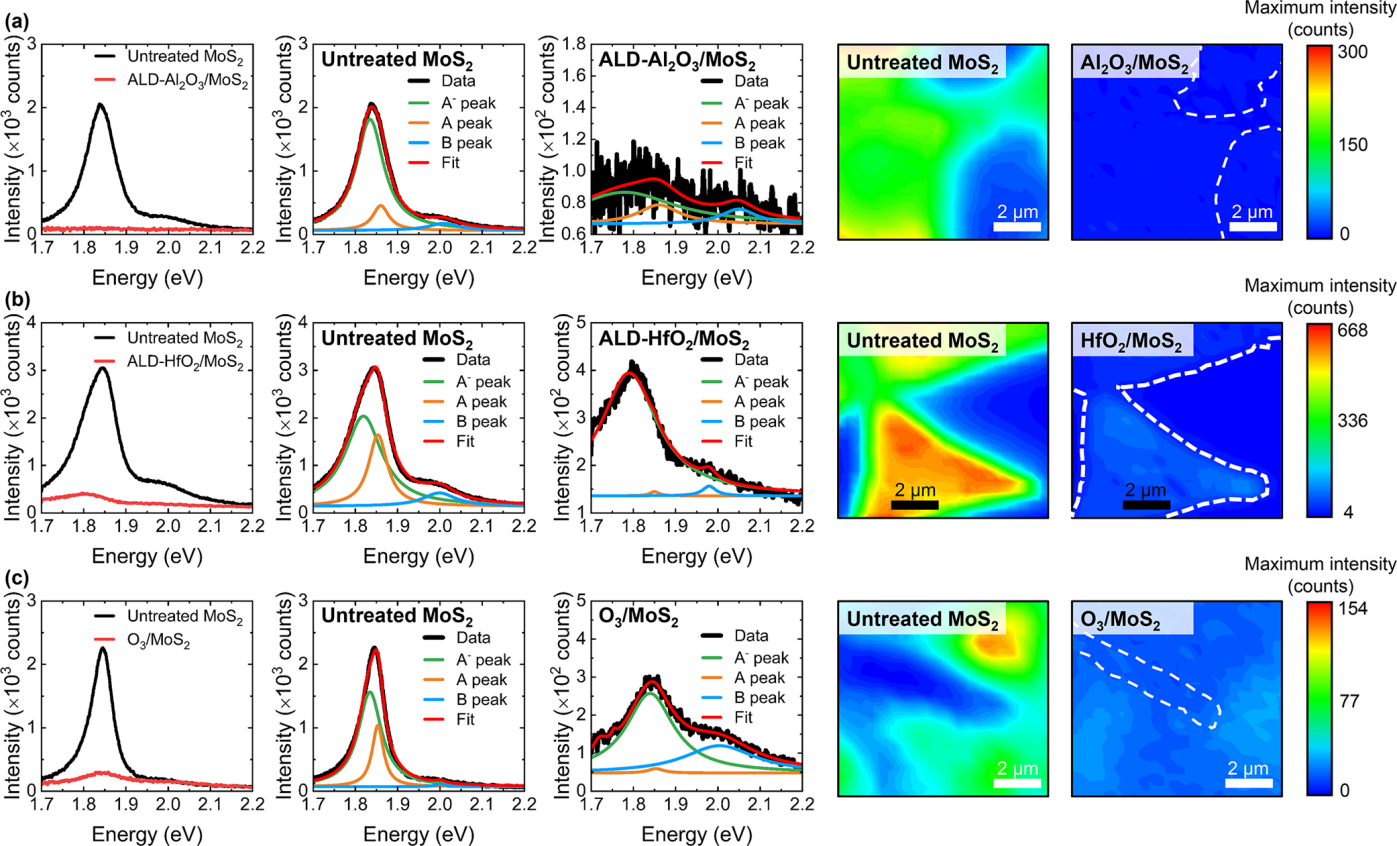
Raw and deconvoluted single-spot PL spectra and maps of the maximum absolute PL intensity before and after (a) O_3_-based ALD of Al_2_O_3_, (b) O_3_-based ALD of HfO_2_, and (c) pulsed O_3_ exposure. Where the MoS_2_ PL intensity is low, white dashed lines outline the MoS_2_ structure.

We explore, via PL and Raman measurements, the impact of each ALD process on the optical properties of 1L MoS_2_. Figures 2 and 3 display single-spot and mapping PL spectra measured from the six different 1L MoS_2_ samples before and after the associated ALD procedure. We have previously verified that the thermal and vacuum effects associated with ALD processes performed using our experimental configuration have little impact on the optical properties of CVD-grown 1L MoS_2_ films.^14^ Therefore, any modifications to the Raman and PL emissions from the 1L MoS_2_ samples studied here can be attributed to either formation of a dielectric film or the ALD process.

The single-spot PL spectra measured here from each of the 1L MoS_2_ samples before ALD processing all broadly exhibit the spectral profile expected from untreated CVD-1L MoS_2_: a superposition of three constituent spectral contributions: A and B exciton transitions and a trion, A^–^, emission.^37^ The dominant emission at ∼1.85 eV arises from radiative recombination of A exciton and A^–^ trion states, and the accompanying shoulder feature at ∼2.0 eV is associated with the B exciton. The variations in the intensity, energy position, and spectral width of the PL signals observed here from the three 1L MoS_2_ samples before ALD processing can be attributed to the spatially inhomogeneous PL character of 1L MoS_2_ films grown via CVD.^38,39^

From Figure 2, it is clear that PEALD of Al_2_O_3_ or HfO_2_ substantially quenches the 1L MoS_2_ PL emission. Similar destruction of the characteristic PL profile is also observed after pulsed O_2_ plasma exposure at 200 °C. The single-site PL spectra measured after each PEALD process are plotted on enlarged axes in Figure S6 of the Supporting Information. In all cases, spatially resolved maps of absolute PL intensity indicate the PL attenuation to be prevalent across each 1L MoS_2_ sample, with the PL signal strength reduced to an intensity comparable to that from the SiO_2_/Si substrate. This agrees well with the findings of Price et al., who reported a significantly attenuated 1L MoS_2_ PL emission following PEALD of HfO_2_.^22^ The authors suggested that the reduction in PL intensity may result from the introduction of defect states during the deposition. Exposing MoS_2_ to O_2_ plasma can create lattice vacancies since energetic O species impact the monolayer surface and displace S atoms in the crystal structure.^30,40^ Regions of molybdenum trioxide (MoO_3_) may then develop at such defect sites via the reaction 2MoS_2_ + 7O_2_ → 2MoO_3_ + 4SO_2_.^40,41^ Kang et al. proposed that incorporation of MoO_3_ in the MoS_2_ lattice yields a direct-to-indirect bandgap transition as the MoO_3_ defect concentration increases, thus suppressing the MoS_2_ PL signature.^40^ Moreover, substantial oxidation of 1L MoS_2_ toward single-layer MoO_3_ would not be expected to emit an appreciable PL signal, since the bandgap of MoO_3_ (3.2 eV)^42^ exceeds the excitation photon energy used here (532 nm, 2.33 eV).^34^

Akin to the effects of PEALD, we find that thermal ALD of Al_2_O_3_ via O_3_ co-reactant heavily attenuates the MoS_2_ PL intensity (Figure 3a). The ALD-Al_2_O_3_/MoS_2_ sample does still emit the characteristic MoS_2_ PL profile, though the intensity is so weak that the spectrum is dominated by noise. However, we find the PL signature of 1L MoS_2_ survives at an appreciable intensity following both O_3_-based ALD of HfO_2_ and pulsed exposure to O_3_. We speculate that the reaction of TMA with O_3_ and O* species at the 1L MoS_2_ surface is more damaging than that of the alkyl amine TDMAH, since TMA is a volatile and aggressive alkyl precursor.^43^

After both ALD of HfO_2_ via O_3_ co-reactant and pulsed O_3_ treatment, an attenuated, red-shifted, and broadened PL emission was observed from 1L MoS_2_, as evident in Figure 3b,c. Maps of the peak PL energy and full width at half-maximum (FWHM) of the PL signal measured from the MoS_2_ samples subjected to O_3_-based ALD of HfO_2_ and pulsed O_3_ exposure are provided in Figure S7 in the Supporting Information. The mapping data confirm the ALD-induced changes to the PL character to be widespread across each MoS_2_ sample. O_3_-based ALD of HfO_2_ was found to reduce the absolute MoS_2_ PL intensity by ∼8×, with a large average redshift of the PL energy (∼58 meV) and ∼2× widening of the FWHM also observed. The pulsed O_3_ treatment gave rise to a smaller average PL attenuation of ∼4×, a downward shift of only ∼11 meV and ∼2× broadening of the spectral line width. This suggests the presence of the HfO_2_ layer introduces further reduction and redshift of the PL signal, beyond the effects of O_3_ exposure. The decreased, red-shifted and broadened PL observed from 1L MoS_2_ after O_3_-based ALD of HfO_2_ and exposure to O_3_ pulses may be indicative of n-type doping effects.^44^ It is known that deposited high-κ films can dope the underlying MoS_2_ via fixed charges at the dielectric/MoS_2_ interface,^23^ while O_3_ has also been shown to alter the electron density in single-layer TMDCs.^45^ To examine further the impact of O_3_-based ALD on the electron density in 1L MoS_2_, we decompose the single-spot PL spectra measured before and after ALD into the A and B excitonic and A^–^ trionic spectral contributions, as shown in Figure 3. The ratio of the absolute intensity of the A^–^ peak to that of the A exciton, A^–^/A, can be related to the charge carrier density in 1L MoS_2_.^44^ We find the A^–^/A ratio increases from ∼1.1 to ∼2.7 following ALD of HfO_2_, and from ∼1.5 to ∼4.3 after pulsed exposure to O_3_. These modifications of the A^–^/A ratio indicate O_3_-based ALD of HfO_2_ and pulsed O_3_ treatment encourage the formation of trions and suggest an increase in the electron density, but further spectroscopic evidence is required.

**Figure 4:**
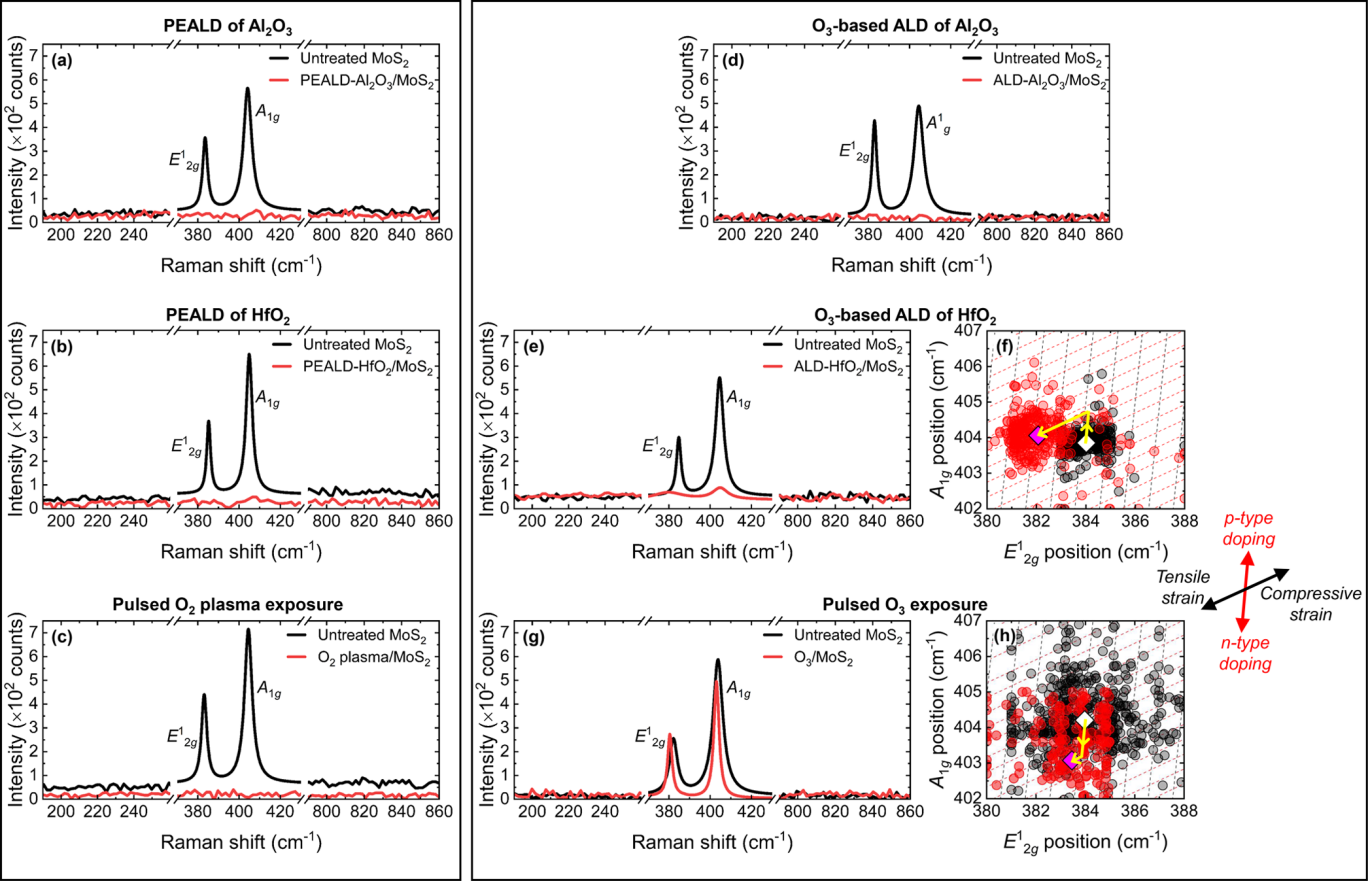
Single-spot Raman spectra obtained from 1L MoS_2_ before and after (a) PEALD of Al_2_O_3_, (b) PEALD of HfO_2_, (c) pulsed O_2_ plasma exposure, (d) O_3_-based ALD of Al_2_O_3_, (e) O_3_-based ALD of HfO_2_, and (g) pulsed O_3_ exposure. Note the broken axes that highlight three key wavenumber ranges. Correlative plots of A_1g_ and E^1^_2g_ peak positions extracted from Raman mapping data acquired from 1L MoS_2_ before (black) and after (red) (f) O_3_-based ALD of HfO_2_, and (h) pulsed O_3_ exposure. The small circular translucent markers arise from every pixel extracted from the Raman mapping data, and the large diamond points indicate the corresponding average values. Strain isolines (black dashed lines) correspond to Δε = ±0.2% variations in the strain, and doping isolines (red dashed lines) indicate relative changes in the electron density of Δ*n* = ±0.2 × 10^13^ cm^–2^. The directions of the strain and doping effects are highlighted.

We utilize Raman spectroscopy to further examine the impact on 1L MoS_2_ of the different ALD processes applied in this work, since the vibrational modes of 1L MoS_2_ are sensitive to external perturbation.^46^ Single-spot Raman spectra were obtained from each 1L MoS_2_ sample before and after ALD and are presented in Figure 4.

We separate each single-site Raman spectrum into three segments to highlight key spectral details. The distinct Raman spectrum of 1L MoS_2_ contains two characteristic peaks: an in-plane E^1^_2g_ mode at ∼384 cm^–1^ and an out-of-plane A_1g_ vibration at ∼403 cm^–1^.^46^ Both features disappear from the Raman spectrum following PEALD of Al_2_O_3_ or HfO_2_, and indeed after pulsed exposure to O_2_ plasma. This removal of the Raman signature indicates that the crystal structure of each 1L MoS_2_ sample is so drastically altered by the exposure to O_2_ plasma that it no longer resembles MoS_2_, consistent with significant oxidation.^47^ The presence of MoO_3_ would be expected to yield a Raman peak at ∼820 cm^–1^, resulting from the symmetric stretching of terminal oxygens.^48^ However, as shown in Figure 4, no modes appear around this wavenumber in any of the Raman spectra measured following the PEALD processes performed here, so there is no evidence for Mo=O bonding in the 1L MoS_2_ samples. A Raman feature at ∼226 cm^–1^ would be indicative of Mo–O bonding,^41^ yet no such peak is seen in any of the Raman spectra obtained here after PEALD. The stoichiometric identity of any MoO_*x*_ in our 1L MoS_2_ samples is therefore unclear, but the presence of MoO_2_ or MoO_3_ reported elsewhere in O_2_ plasma-treated MoS_2_ is not evidenced by our Raman data.^23,40,41,47^ It has been suggested that partial oxidation of MoS_2_ may yield regions of molybdenum oxysulfide (MoS_*x*_O_*y*_) or the formation of molybdenum sulfates (Mo(SO_4_)_*z*_).^30,34^ Incomplete oxidation of 1L MoS_2_ following the PEALD processes applied in this work may be somewhat surprising, since each sample was subjected to 200 pulses of 6 s of exposure to O_2_ plasma, equivalent to 20 min of O_2_ plasma treatment. However, each pulse of O_2_ plasma was succeeded by a ∼4 s Ar purge step that likely influenced the cumulative nature of the oxidation. Further compositional analysis is therefore required to establish the oxidation process that occurs in 1L MoS_2_ upon treatment with O_2_ plasma in our PEALD configuration.

From the single-site Raman spectra in Figure 4d, the 1L MoS_2_ Raman signature is removed following ALD of Al_2_O_3_ via O_3_ co-reactant. This, coupled with the sizable attenuation of the PL signal, suggests O_3_-based ALD of Al_2_O_3_ damages the underlying 1L MoS_2_ structure. This deposition does yield an ALD-Al_2_O_3_ film with ∼56% coverage as observed via AFM and XPS characterization, but the optical properties of CVD-1L MoS_2_ are depleted during the process. In contrast, the characteristic MoS_2_ Raman signal is maintained after O_3_-based ALD of HfO_2_ and after pulsed O_3_ treatment. Maps of the intensities, positions, and FWHMs of the characteristic MoS_2_ Raman peaks measured before and after these processes are provided in Figures S8 and S9 in the Supporting Information. While the E^1^_2g_ and A_1g_ Raman modes do indeed survive ALD of HfO_2_ via O_3_ co-reactant, the intensities are reduced by up to ∼7×. The characteristic Raman peak separation was also seen to increase from ∼19.8 to ∼22.0 cm^–1^ following O_3_-based ALD of HfO_2_, exceeding the expected value for 1L MoS_2_,^46^ and we estimate the FWHM of the E^1^_2g_ and A_1g_ Raman modes to be increased by a factor of ∼2. These changes to the Raman spectrum indicate some modification of the MoS_2_ crystal symmetry may have occurred during ALD. In comparison, the intensity of the 1L MoS_2_ Raman signal is virtually unchanged by pulsed exposure to O_3_. Since only small modifications of the peak separation and FWHMs were also observed, it is implied that the impact of pulsed O_3_ treatment on the MoS_2_ crystal structure is not as harsh as that of the HfO_2_ deposition.

The in-plane E^1^_2g_ and out-of-plane A_1g_ MoS_2_ peaks are known to be sensitive to strain and electron density, respectively.^49^ Hence, shifts in positions of these modes can reveal the impact of a treatment on the strain and doping in MoS_2_. To quantify changes in electron density, *n*, and biaxial mechanical strain, ε, induced in 1L MoS_2_ following ALD of HfO_2_ and pulsed O_3_ treatment, we perform a correlative analysis of the characteristic Raman peaks in the mapping data. This process is commonly applied to graphene^50^ and MoS_2_ systems,^51^ and is detailed in Section S7 of the Supporting Information. Correlative plots of the A_1g_ wavenumbers as a function of the E^1^_2g_ peak positions extracted from the mapping data for the O_3_-based ALD of HfO_2_ and pulsed O_3_ exposure are displayed in Figure 4f,g, respectively, each with an overlaid ε–*n* coordinate grid. Doping isolines are represented by the red dashed lines and correspond to Δ*n* = ±0.2 × 10^13^ cm^–2^ variations in the electron density. Δ*n* > 0 indicates n-type doping, and p-type doping is represented by Δ*n* < 0. The dashed black lines outline the strain isolines and indicate relative changes in the strain of Δε = ±0.2%, where Δε > 0 signifies tensile strain and Δε < 0 implies compressive strain.

A negligible ALD-induced redshift in the A_1g_ peak position was evident in the single-spot Raman spectra after O_3_-based HfO_2_ growth, yet the mapping data reveals a more reliable average blueshift of ∼0.2 cm^–1^. The Raman mapping analysis also indicates the E^1^_2g_ vibration experiences a relatively large redshift of ∼1.9 cm^–1^ following ALD of HfO_2_. After pulsed O_3_ treatment, the E^1^_2g_ mode in 1L MoS_2_ is red-shifted by ∼0.5 cm^–1^ on average, with an accompanying average redshift in the position of the A_1g_ of ∼1.1 cm^–1^ peak. We estimate ALD of HfO_2_ via O_3_ co-reactant reduces the electron density in 1L MoS_2_ by ∼0.4 × 10^13^ cm^–2^ and imparts tensile strain (Δε ∼ 0.3%). The changes in the Raman spectra upon exposure to O_3_ pulses correspond to an increase in the electron density of ∼0.5 × 10^13^ cm^–2^ and the introduction of a comparatively small degree of tensile strain (Δε < 0.1%). The Raman mapping data therefore indicate O_3_-based ALD of HfO_2_ p-type dopes 1L MoS_2_, whereas an opposite n-type doping effect results from pulsed O_3_ treatment. The apparent p-type doping of 1L MoS_2_ by O_3_-based HfO_2_ growth is expected from the oxygen-rich stoichiometry estimated via XPS^23^ but is inconsistent with the increased A^–^/A ratio evaluated in our earlier analysis of the PL spectra. However, the greater shift in the position of the E^1^_2g_ peak compared to that of the A_1g_ mode following ALD of HfO_2_ via O_3_ reactant implies that strain effects, rather than charge doping, dominate the HfO_2_/MoS_2_ interfacial interaction.^46^ The attenuated and red-shifted PL spectrum, and increased A^–^/A ratio, observed from 1L MoS_2_ after O_3_-based ALD of HfO_2_ can be explained by the significant increase in tensile strain due to the presence of the HfO_2_ film. The increased tensile strain in the ALD-HfO_2_/1L MoS_2_ structure likely originates from the lattice mismatch between the MoS_2_ and the deposited HfO_2_.^22^ The introduction of tensile strain in 1L MoS_2_ has been shown to modify the band structure such that the direct bandgap becomes indirect, and its energy reduces.^52−55^ As a result, the PL emission is attenuated and red-shifted. Moreover, the altered band structure facilitates the accumulation of free electrons, and trion formation is encouraged, in agreement with our observed increase in the A^–^/A ratio.^56,57^

Conversely, pulsed O_3_ treatment imparts only a small degree of tensile strain in 1L MoS_2_, implying n-type charge doping is the primary effect of this process. We attribute the changes to the 1L MoS_2_ PL spectra (attenuation, redshift, broadening, and increased A^–^/A ratio) observed following pulsed O_3_ exposure to this n-type doping.^44,58^ The increased electron density in O_3_-exposed 1L MoS_2_ may arise from the generation of defects and/or the incorporation of O atoms in the MoS_2_ lattice.^10,59^ The anticipated benefits of a highly oxidizing co-reactant such as O_3_ arise from an improved reaction between the precursor and the co-reactant. The interaction between the strong oxidant and the MoS_2_ surface must also be considered and O_3_ exposure may oxidize 1L MoS_2_, thus degrading its optical behavior.^11,60^ However, unlike after the O_2_ plasma treatment discussed earlier, the 1L MoS_2_ Raman signal is preserved following the pulsed O_3_ process. Hence, we suggest that O_3_ exposure partially oxidizes the monolayer surface such that the characteristic PL and Raman signals are modified but not completely removed. Despite suspected oxidation of the MoS_2_, no peaks at ∼225 or ∼820 cm^–1^ are observed in the Raman spectrum following any of the O_3_-based ALD processes, so no presence of crystalline MoO_*x*_ is explicitly apparent in the Raman data. The apparent increased oxidation of 1L MoS_2_ following PEALD with O_2_ plasma compared to that observed after ALD via O_3_ co-reactant may be attributed to the presence of relatively high energy species in the O_2_ plasma despite the remote delivery but also the increased exposure to O-containing species. In the ALD process that applied the pulsed O_3_ treatment, the duration of each O_3_ pulse was only 0.15 s, much shorter than the 6 s of O_2_ plasma pulsed in the corresponding O_2_ plasma recipe. The total time of exposure to O_3_ following 200 pulses was only 30 s, compared to 20 min of O_2_ plasma exposure.

### 3.3 XPS Characterization of 1L MoS_2_ Exposed to O_2_ Plasma and O_3_

**Figure 5:**
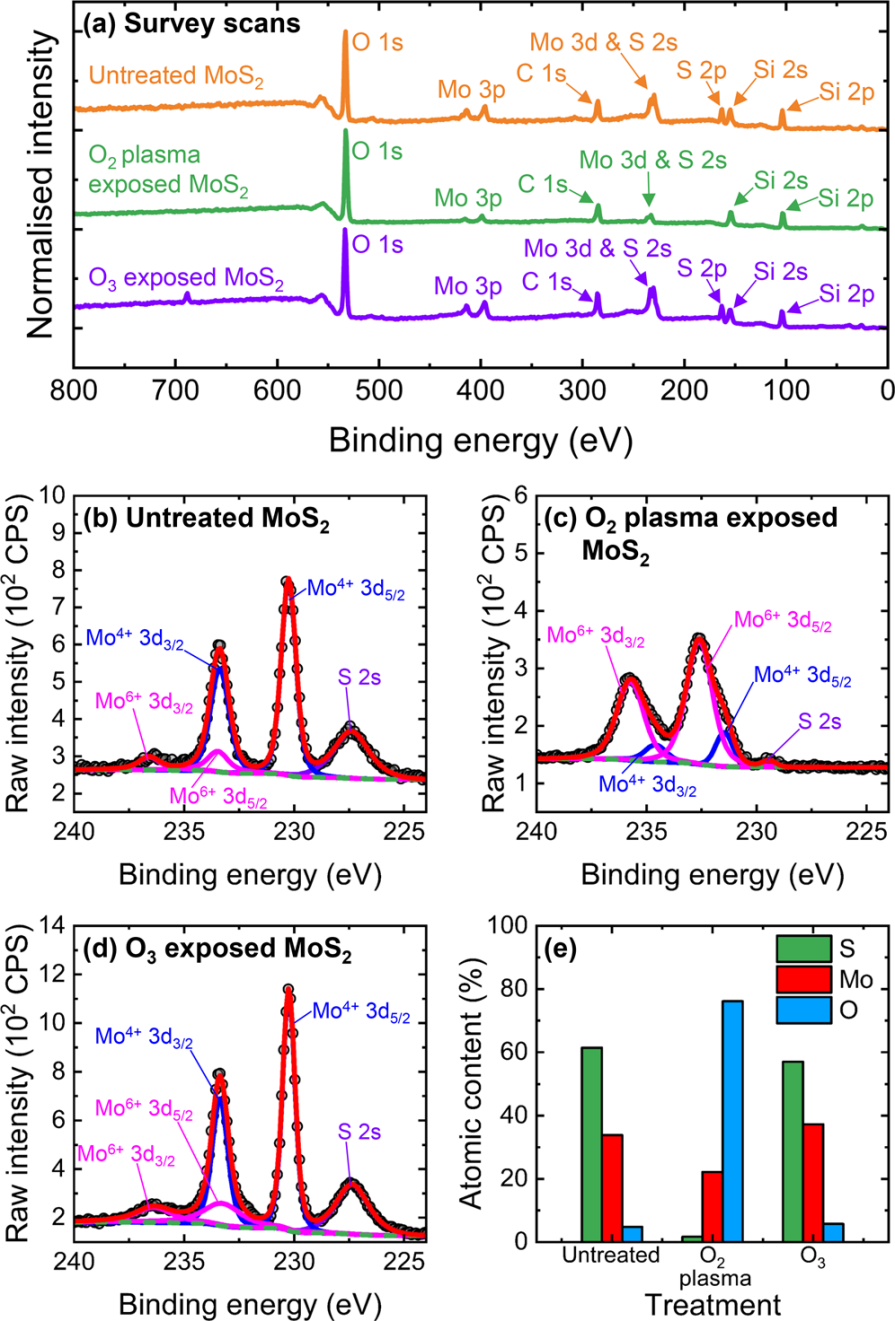
(a) XPS survey scans from untreated, O_2_ plasma exposed, and O_3_ exposed 1L MoS_2_ samples. The binding energies in all the XPS spectra were calibrated to the C 1s core level and the constituent peaks are labeled. High-resolution, deconvoluted XPS spectra in counts per second (CPS) of the Mo 3d core level regions from the (b) untreated, (c) O_2_ plasma exposed, and (d) O_3_ exposed 1L MoS_2_ samples. The black circular markers represent the raw, recorded data, and the solid, colored lines correspond to fitted data. The Shirley background is indicated by the dashed green line. (e) Comparison of the relative atomic content of Mo, S and O in the untreated, O_2_ plasma exposed, and O_3_ exposed 1L MoS_2_ samples as extracted from the XPS data.

XPS was performed to elucidate a deeper understanding of the changes to the crystal structure of 1L MoS_2_ that result from exposure to O_2_ plasma and O_3_ in the ALD processes studied here. XPS data were collected from the 1L MoS_2_ samples subjected to 200 cycles of pulsed O_2_ plasma exposure and pulsed O_3_ treatment, with XPS measurements also performed on a separate untreated 1L MoS_2_ sample. Selected XPS are presented in Figure 5.

From the survey spectra displayed in Figure 5a, we find the pulsed O_2_ plasma treatment at 200 °C results in an apparent loss of S, with no S 2p peak present. This is further evidenced by the high-resolution scan of the Mo 3d core level region in Figure 5c, where the intensity of the adjacent S 2s peak in the 1L MoS_2_ sample exposed to pulsed O_2_ plasma is significantly quenched compared to in the untreated sample in Figure 5b. There is little change to the abundance of S in 1L MoS_2_ following pulsed O_3_ treatment, with the intensity of the S 2s contribution comparable between the untreated and O_3_-treated samples. Deconvolution of the Mo 3d spectrum obtained from the untreated sample reveals the signature MoS_2_ spin–orbit doublet associated with the Mo^4+^ oxidation state typical of the Mo–S bond: Mo 3d_5/2_ and Mo 3d_3/2_ features at 230.3 and 233.4 eV, respectively.^61,62^ A second pair of less intense Mo 3d_5/2_ and Mo 3d_3/2_ peaks was observed at higher binding energies (233.5 and 236.6 eV, respectively) and attributed to the Mo^6+^ oxidation state. Representing an oxidation of Mo^4+^, the Mo^6+^ state indicates the presence of MoO_3_.^63^ The detection of Mo^6+^ in untreated synthetic 1L MoS_2_ is likely a result of residual oxide (<15% of the total Mo content) that forms during or after the CVD growth.^10,34,64^ The Mo^4+^ and Mo^6+^ peaks are relatively unchanged in the 1L MoS_2_ sample that was subjected to the pulsed O_3_ treatment. However, we find a reduced contribution of the Mo^4+^ doublet in the 1L MoS_2_ sample following 200 cycles of pulsed O_2_ plasma exposure, and the relative intensity of the Mo^6+^ peaks is considerably enhanced such that ∼86% of the total Mo content is in MoO_3_. Probing the O 1s core level can also reveal the presence of MoO_*x*_ in 1L MoS_2_ and high-resolution XPS spectra measured from the untreated, plasma-exposed, and O_3_-treated samples are provided in Figure S11a to c in the Supporting Information. All samples exhibit a dominant peak at ∼533 eV that arises from the SiO_2_ substrate and C–O contamination, accompanied by a much weaker organic contaminant C=O signal at ∼532 eV. We also observe a small feature at ∼530.9 eV that can be assigned to Mo–O in MoO_3_ structures.^64,65^ The relative contribution of this Mo–O peak to the total O 1s XPS signal is more significant in the plasma-processed 1L MoS_2_ sample, corroborating the treatment-induced increase in the oxide content. From our core level XPS spectra, we cannot exclude the presence of MoO_2_ in any of the 1L MoS_2_ samples. Since the binding energies of the Mo^4+^ doublet associated with MoO_2_ overlap with those characteristic of MoS_2_, it is challenging to identify MoO_2_ solely via Mo 3d XPS spectra when MoS_2_ and MoO_3_ are present in the material system.^41^

The formation of MoO_3_ during O_2_ plasma treatment is explicitly indicated by the XPS data is not captured in the Raman spectra presented in Figure 4. We suggest that the absence of MoO_3_ peaks in the Raman spectra measured from the 1L MoS_2_ samples following pulsed O_2_ plasma exposure or PEALD of Al_2_O_3_ or HfO_2_ may be due to the amorphous nature of the MoO_3_ that is formed.^34^ MoO_3_ can exist as a number of polymorphs, and the only thermodynamically stable phase is orthorhombic α-MoO_3_,^66^ which exhibits a layered structure and has a distinct Raman signature at room temperature. The amorphous phase of MoO_3_ is known to be the most unstable, with monoclinic β-MoO_3_ and hexagonal h-MoO_3_ exhibiting metastability.^67^ Exposing MoS_2_ to a remote O_2_ plasma at 200 °C continuously for 20 min has been shown to form a uniform, amorphous surface layer of MoO_3_ that has no long-range crystallinity.^29,68^ The Raman spectrum of amorphous MoO_3_ can differ from that of α-MoO_3_ and may explain why no MoO_3_ features were detected in the Raman spectra collected from the 1L MoS_2_ samples following PEALD treatment.^34,69^ Alternatively, the weaker surface sensitivity of Raman spectroscopy compared to XPS may prevent detection of a thin MoO_3_ layer on MoS_2_.^29^

Finally, we utilize the integrated intensities from XPS core level spectra data to estimate the relative abundances of Mo, S, and O in each sample. Figure 5e compares the elemental composition of the untreated, plasma-exposed, and O_3_-treated 1L MoS_2_ samples. We determine the atomic ratio of S/Mo to be ∼1.8 in untreated 1L MoS_2_, ∼1.5 in O_3_-exposed 1L MoS_2_ but only ∼0.1 in the plasma-treated sample, confirming the desulfurization of MoS_2_ during exposure to O_2_ plasma. No S 2p signal was detected in 1L MoS_2_ following O_2_ plasma treatment, but S 2p core level spectra obtained from the untreated and O_3_-treated 1L MoS_2_ samples are displayed in Figure S11d,e, respectively. While significant loss of S does not occur in 1L MoS_2_ after pulsed exposure to O_3_, oxidation of S atoms to form SO_2_ and other substoichiometric sulfur oxides (SO_*x*_) is apparent.^11,70^ We conclude from the XPS data that the O_2_ plasma process applied here substantially oxidizes 1L MoS_2_ to MoO_3_, thus depleting the S content. No such considerable oxidation of MoS_2_ to MoO_3_ results from O_3_ exposure, with only partial oxidation of S to SO_2_ and SO_*x*_ detected.

## 4 Conclusions

We have systematically studied two different co-reactants for direct ALD of high-κ dielectric films on CVD-1L MoS_2_: O_2_ plasma and O_3_. We have verified that exposure to remote O_2_ plasma during PEALD of Al_2_O_3_ or HfO_2_ at 200 °C significantly oxidizes synthetic 1L MoS_2_ to MoO_3_, thus degrading the intrinsic material structure and removing its characteristic optical behavior. Conversely, we find exposing 1L MoS_2_ to O_3_ during an ALD process does not oxidize 1L MoS_2_ to the same extent, promoting O_3_-based ALD as a route for the realization of high-κ dielectrics on 1L MoS_2_ for nanoscale FET device fabrication. We have demonstrated direct growth on CVD-1L MoS_2_ of a relatively smooth (RMS roughness ∼0.4 nm) and oxygen-rich HfO_2_ film with ∼90% surface coverage via thermal ALD with O_3_ co-reactant at 200 °C. Single-site and mapping Raman and PL measurements reveal this oxygen-rich ALD-HfO_2_ film p-type dopes 1L MoS_2_, while also introducing tensile strain, thus opening the possibility of electron density modulation and strain engineering of CVD-1L MoS_2_ via O_3_-based ALD of high-κ dielectrics.

## Data Availability

Data underpinning figures in this paper can be freely downloaded from https://wrap.warwick.ac.uk/190565/. Requests for additional data should be made directly to the corresponding authors.

## Supporting Information Available

AFM images and line profiles of untreated 1L MoS_2_ films (Figure S1), Raman peak separation mapping data for untreated 1L MoS_2_ films (Figure S2), AFM images of areas for RMS roughness analysis (Figure S3), XPS data for PEALD of Al_2_O_3_ and HfO_2_ (Figure S4), line profiles from O_2_ plasma- and O_3_-treated 1L MoS_2_ (Figure S5), enlarged single-spot PL of post-PEALD 1L MoS_2_ (Figure S6), further PL mapping data for O_3_-based ALD processes (Figure S7), Raman mapping data for O_3_-based ALD processes (Figures S8–S9), correlative analysis of strain and doping in monolayer MoS_2_ (Figure S10), and additional XPS data for untreated, O_2_ plasma-treated, and O_3_-treated 1L MoS_2_ (Figure S11) (PDF)
