# Supplementary material for: Impact of Co-Reactants in Atomic Layer Deposition of High-κ Dielectrics on Monolayer Molybdenum Disulfide
Source: ACS Appl Nano Mater. 2025 Apr 1;8(14):7334–46. doi: 10.1021/acsanm.5c00901 (PMC11997949; doi:10.1021/acsanm.5c00901)
Supplement: Supplementary file 1 — an5c00901_si_001.pdf [file an5c00901_si_001.pdf]

Supporting Information

Impact of Co-reactants in Atomic Layer Deposition of High-κ Dielectrics on Monolayer Molybdenum Disulfide

Brendan F. M. Healy[1,], Sophie L. Pain[1], Marc Walker[2], Nicholas E. Grant[1], and John D. Murphy[1,]**

[1]School of Engineering, University of Warwick, Coventry, CV4 7AL, United Kingdom

[2]Department of Physics, University of Warwick, Coventry, CV4 7AL, United Kingdom

* Corresponding Authors

Brendan F. M. Healy - Email: brendan.f.m.healy@warwick.ac.uk

John D. Murphy - Email: john.d.murphy@warwick.ac.uk

Contents

1. Verification of 1L MoS_2 .... S-1

2. AFM images of areas for RMS roughness analysis S-3

3. XPS data for PEALD and O_3-based ALD of Al_2O_3 and HfO_2 .... S-4

4. Line profiles from O_2 plasma- and O_3-treated 1L MoS_2 .... S-5

5. Enlarged single-spot PL of post-PEALD 1L MoS_2 .... S-6

6. Further PL mapping data for O_3-based ALD processes S-7

7. Raman mapping data for O_3-based ALD processes S-8

8. Correlative analysis of strain and doping in 1L MoS_2 .... S-10

9. Additional XPS data for untreated, O_2 plasma-treated, and O_3-treated 1L MoS_2 .... S-13

References S-14

1. Verification of 1L MoS_2

We verify the single-layer nature of the as-received chemical vapour deposited (CVD)-monolayer molybdenum disulfide (1L MoS_2) films *via* atomic force microscopy (AFM) and Raman spectroscopy.

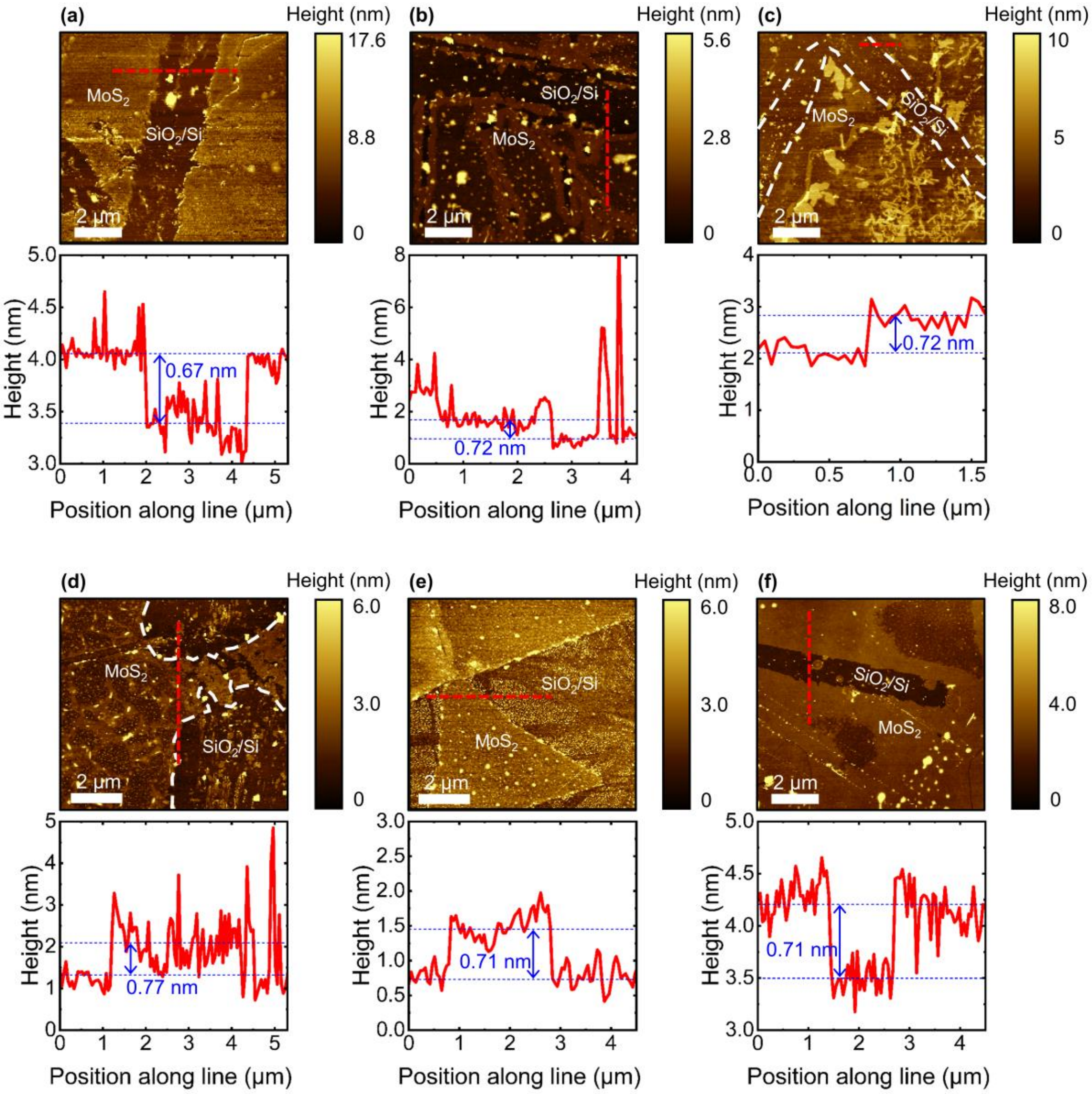

Figure S1. (a) to (f) AFM images of each untreated 1L MoS_2 film used in this study, with associated line profiles given below each image. The dashed red line indicates the line from which the profile was extracted.

Figure S1 presents low-magnification topographic AFM images of the untreated CVD-1L MoS_2 films. We trace height profiles across the MoS_2 films and their boundaries with the silicon dioxide/silicon (SiO_2/Si) substrate. Any surface contaminant features of significant height are excluded from the step height estimation, and we obtain step heights of ~0.7 nm in each case, in excellent agreement with the expected thickness of CVD-1L MoS_2.[1-3] We utilise Raman spectroscopy further to confirm the single-layer nature of the MoS_2 films. 1L MoS_2 exhibits a distinct Raman signature comprising two characteristic modes: an in-plane $E^1{}_{2g}$ vibration at ~384 cm^{-1} and an out-of-plane A_{1g} feature at ~403 cm^{-1}.[4] The separation between these two peaks serves as a robust indicator of monolayer thickness, typically ~18-21 cm^{-1}.[5, 6] Maps of the Raman peak separation measured from each MoS_2 film prior to any treatment are presented in Figure S2, verifying the presence of 1L MoS_2.

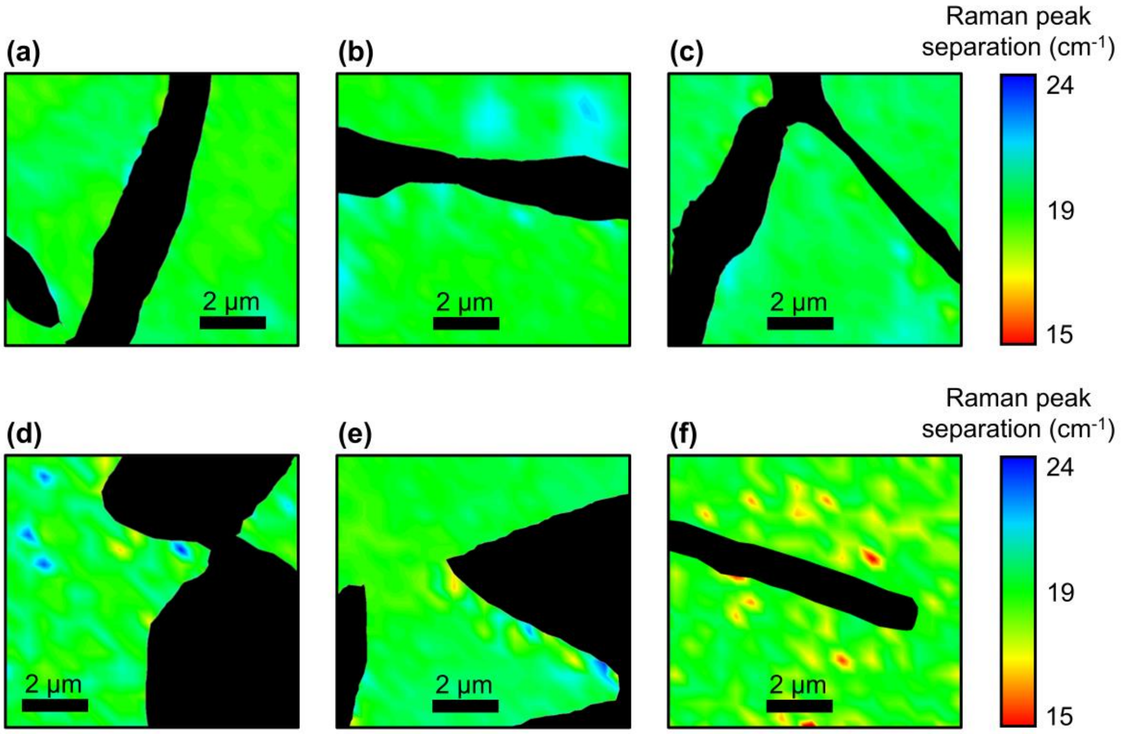

Figure S2. (a) to (f) Maps of the Raman peak separation measured from the untreated CVD-1L MoS_2 films used in this study. The black areas represent the SiO_2/Si substrate where no MoS_2 Raman peaks are detected.

2. AFM images of areas for RMS roughness analysis

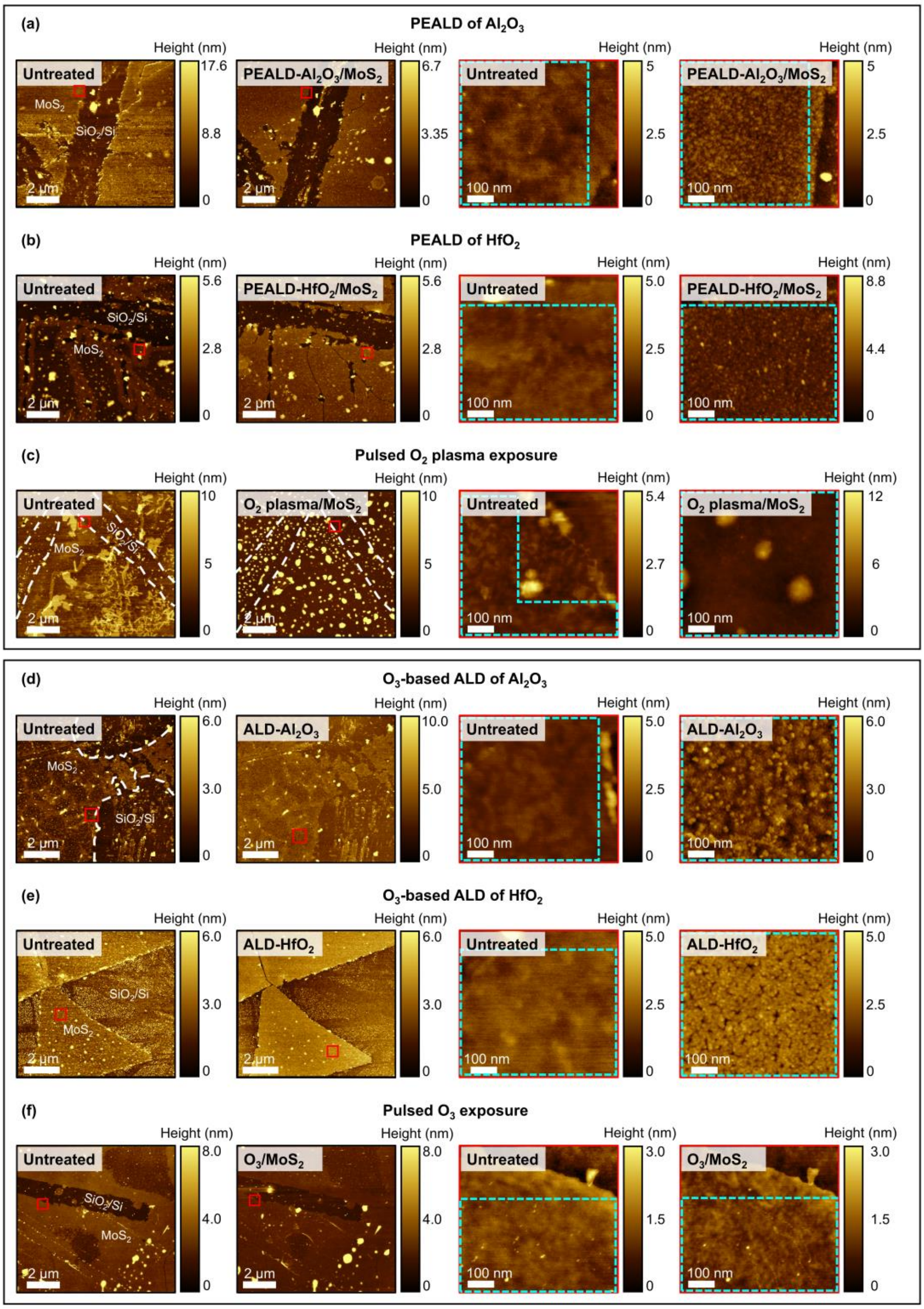

Figure S3. Low- and high-magnification AFM images for PEALD of (a) Al_2O_3 and (b) HfO_2, (c) pulsed O_2 plasma exposure, O_3-based ALD of (d) Al_2O_3 and (e) HfO_2, and (f) pulsed O_3 exposure. Red squares highlight the areas from which high-magnification images were acquired, and cyan dashed regions indicate the areas used for RMS roughness analysis.

3. XPS data for PEALD and O_3-based ALD of Al_2O_3 and HfO_2

Figure S3 presents additional X-ray photoelectron spectroscopy (XPS) data from the aluminium oxide (Al_2O_3) and hafnium dioxide (HfO_2) films grown on the different 1L MoS_2 samples *via* plasma-enhanced atomic layer deposition (PEALD) and ozone (O_3)-based ALD.

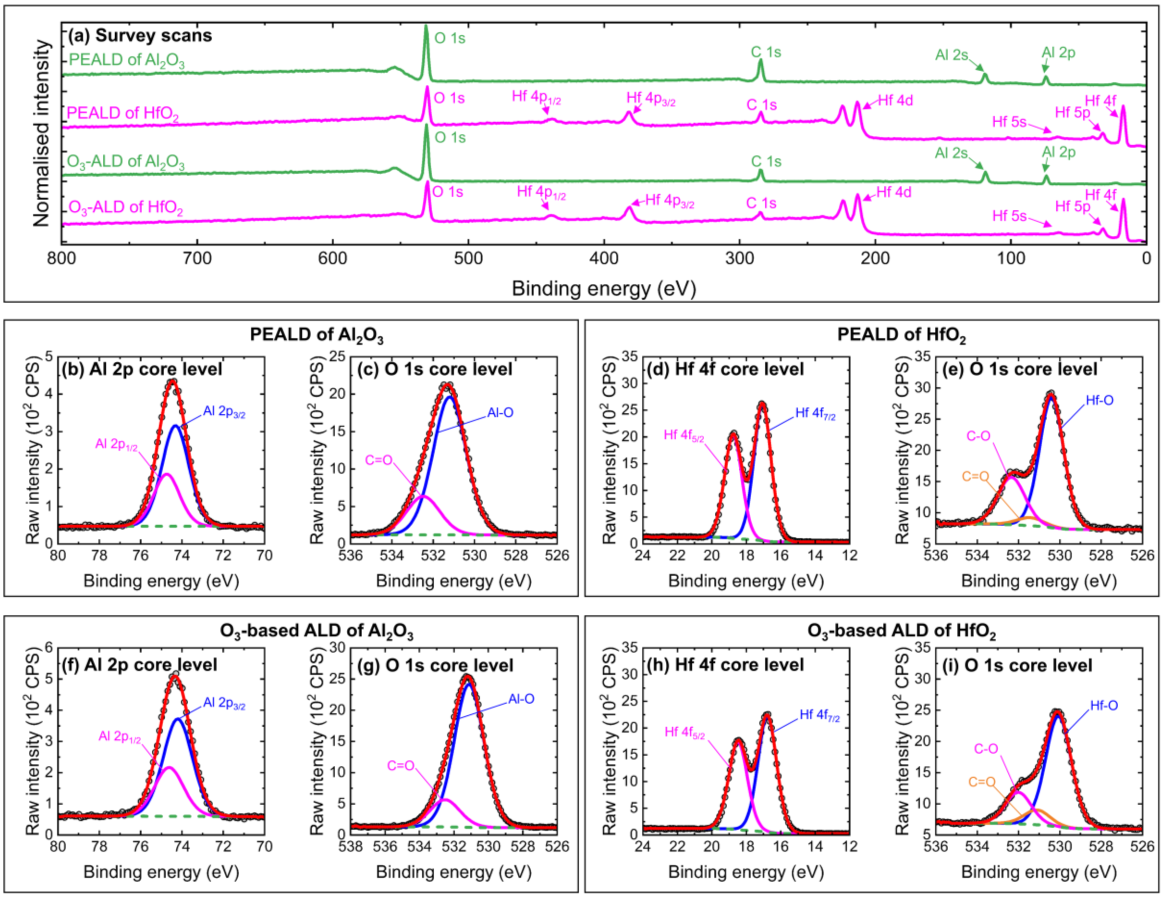

Figure S4. (a) XPS survey scans from the Al_2O_3 and HfO_2 films grown on 1L MoS_2 *via* PEALD and O_3-based ALD. All XPS spectra were calibrated to the C 1s core level and the constituent peaks are labelled. (b)-(i) High-resolution, deconvoluted XPS spectra of the Al 2p, Hf 4f, and O 1s core levels measured from the Al_2O_3 and HfO_2 films. Raw intensities are plotted in counts per second (CPS). The black circular markers represent the raw, recorded data and the solid, coloured lines correspond to fitted data. All peaks in this figure were fitted to Shirley backgrounds, with the exception of (f), where a linear background was used. The background in each spectrum is indicated by the dashed green line.

4. Line profiles from O_2 plasma- and O_3-treated 1L MoS_2

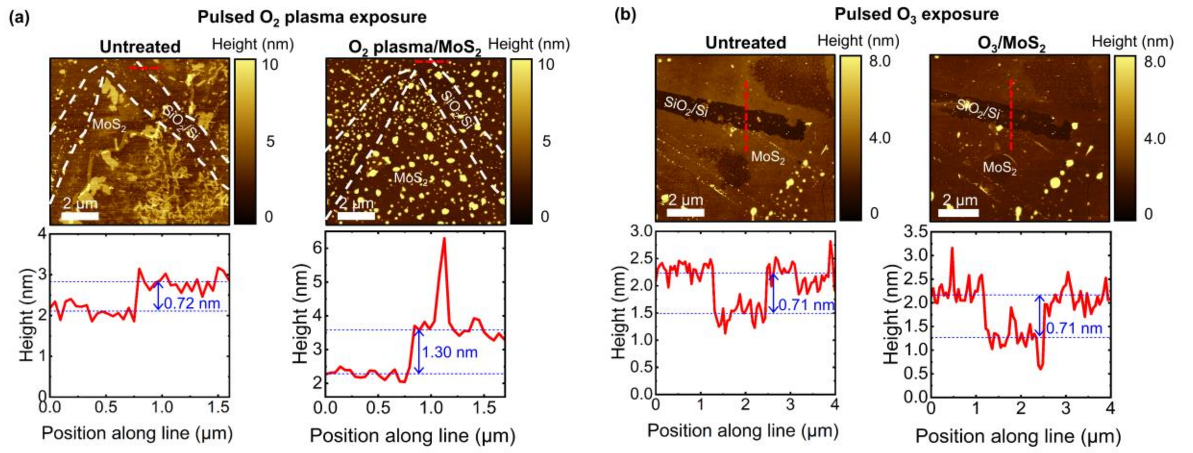

Figure S5. Line profiles taken from the low-magnification images of 1L MoS_2 before and after pulsed exposure to (a) O_2 plasma and (b) O_3. The dashed red line indicates the line from which the profile was extracted. Any surface contaminant features of significant height are excluded from the step height estimation.

5. Enlarged single-spot PL of post-PEALD 1L MoS_2

Figure S6 presents enlarged single-spot photoluminescence (PL) spectra measured from the 1L MoS_2 samples after each PEALD process, taken from Figure 2 in the main text.

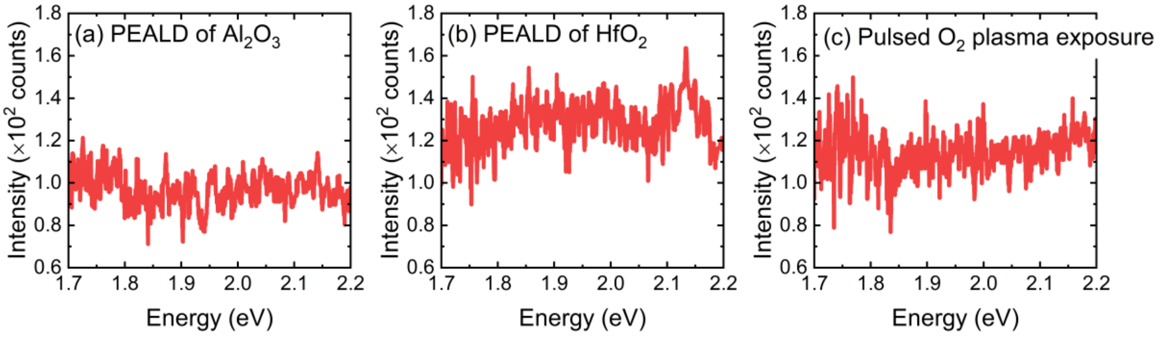

Figure S6. Single-spot PL spectra measured from 1L MoS_2 following (a) PEALD of Al_2O_3, (b) PEALD of HfO_2, and (c) pulsed O_2 plasma exposure.

6. Further PL mapping data for O_3-based ALD processes

Maps of the peak PL energy and full width at half maximum (FWHM) of the PL emission measured from each 1L MoS_2 sample before and after O_3-based ALD of HfO_2 and pulsed exposure to O_3 are shown in Figure S7.

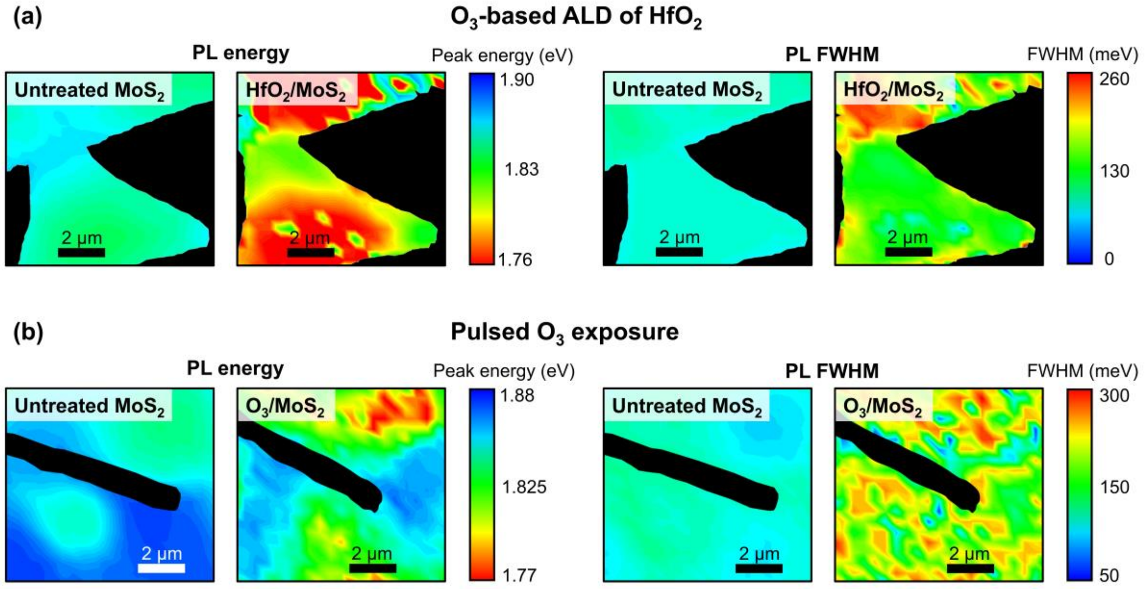

Figure S7. Maps of the peak PL energy and FWHM of the PL signal emitted from each 1L MoS_2 sample before and after (a) O_3-based ALD of HfO_2 and (b) pulsed exposure to O_3. The black areas represent the SiO_2/Si substrate where no MoS_2 PL signal is detected.

7. Raman mapping data for O_3-based ALD processes

Maps of the intensities, positions and linewidths of the characteristic E^1_{2g} and A_{1g} Raman modes peaks measured from 1L MoS_2 samples following ALD of HfO_2 *via* O_3 co-reactant and pulsed exposure to O_3 are shown in Figures S8 and S9.

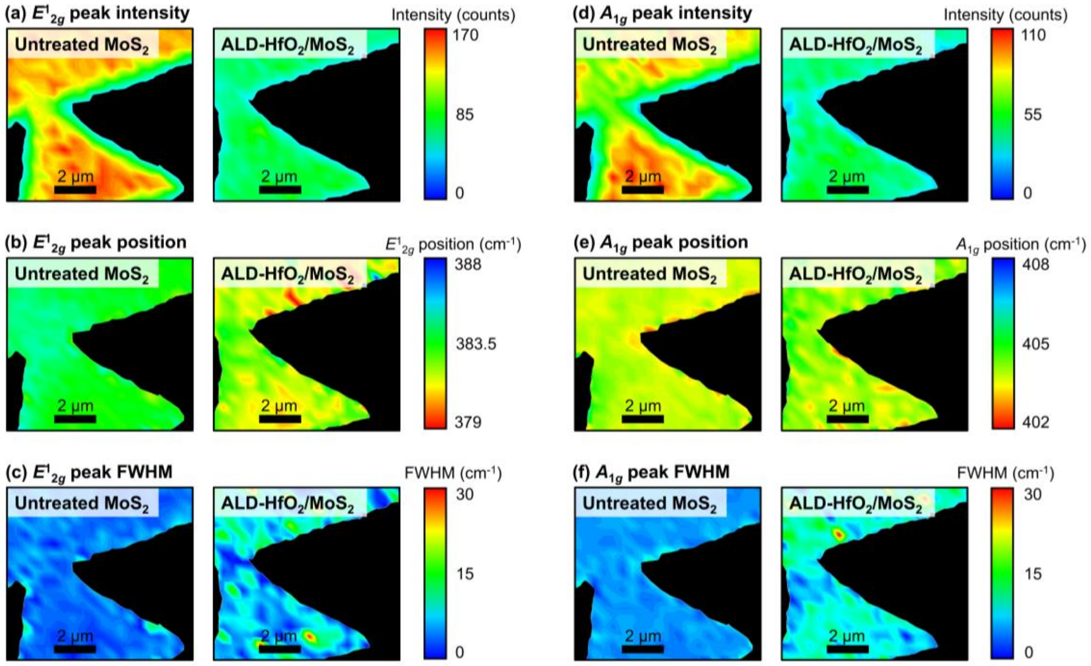

Figure S8. Maps of the intensity, position and FWHM of the (a)-(c) E^1_{2g} peak and (d)-(f) A_{1g} peak measured from 1L MoS_2 before and after ALD of HfO_2 *via* O_3 co-reactant. The black areas represent the SiO_2/Si substrate where no MoS_2 Raman peaks are detected.

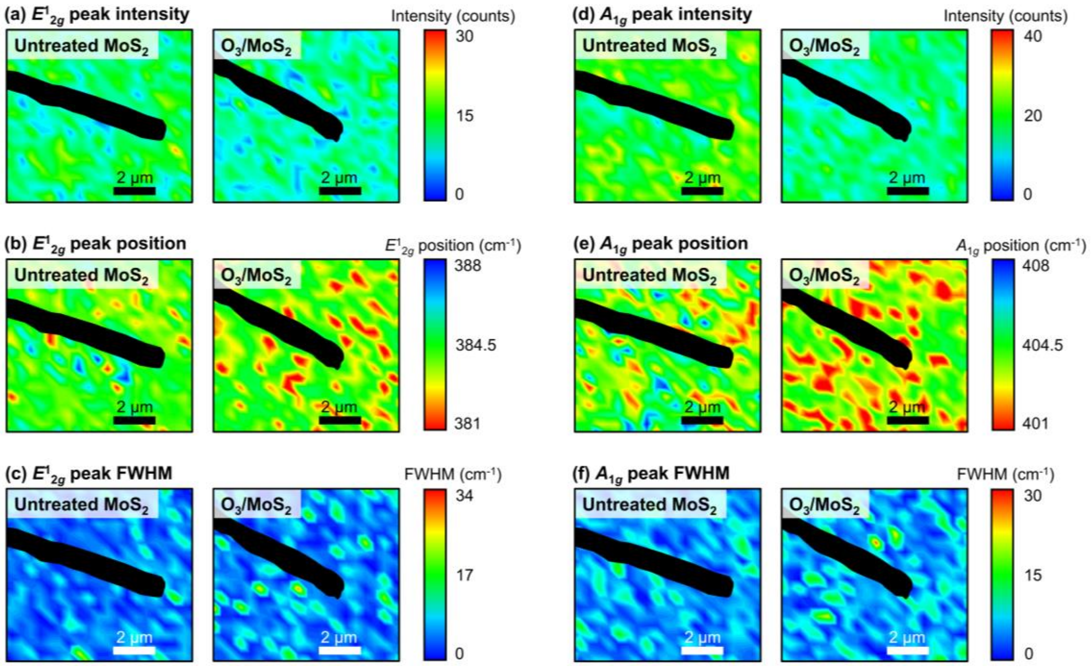

Figure S9. Maps of the intensity, position and FWHM of the (a)-(c) E^1_{2g} peak and (d)-(f) A_{1g} peak measured from 1L MoS_2 before and after pulsed exposure to O_3 in the ALD chamber. The black areas represent the SiO_2/Si substrate where no MoS_2 Raman peaks are detected.

8. Correlative analysis of strain and doping in 1L MoS_2

The following analysis has been previously performed by other authors for graphene [7-11] and more recently 1L MoS_2.[12-17] A freestanding MoS_2 monolayer is a good approximation of an ideal strain-free and undoped system. The characteristic E^1_{2g} and A_{1g} Raman modes for suspended CVD-synthesized 1L MoS_2 appear at ~385 cm^{-1} and ~405 cm^{-1}, respectively,[14, 18] and are denoted $\omega^0_{E'}$ and $\omega^0_{A'}$. The positions of the E^1_{2g} and A_{1g} peaks in the Raman spectrum of a strained and doped 1L MoS_2, $\omega_{E'}$ and $\omega_{A'}$, can be written in terms of the induced biaxial strain, ε, and electron concentration, n:[14]

$$\omega_{E'} = \omega^0_{E'} - 2\gamma_{E'}\omega^0_{E'}\varepsilon + k_{E'}n \quad \text{(S1)}$$

$$\omega_{A'} = \omega^0_{A'} - 2\gamma_{A'}\omega^0_{A'}\varepsilon + k_{A'}n \quad \text{(S2)}$$

where $\gamma_{E'}$ and $\gamma_{A'}$ are the Grüneisen parameters for the two characteristic Raman peaks in 1L MoS_2, and $k_{E'}$ and $k_{A'}$ are the corresponding shift rates of the vibrational modes as a function of carrier concentration, n. The relevant values used in this analysis are detailed in Table S1.

Table S1. Literature values for the Grüneisen parameters and Raman mode shift rates used in the correlative Raman analysis described in this work.

Parameter	Value	Reference
$\gamma_{E'}$	0.84	Rice *et al.* [19]
$\gamma_{A'}$	0.15	Rice *et al.* [19]
$k_{E'}$ ($\times 10^{-13}$ cm)	-0.33	Chakraborty *et al.* [20]
$k_{A'}$ ($\times 10^{-13}$ cm)	-2.22	Chakraborty *et al.* [20]

It follows from Equations S1 and S2, that any shifts in the positions of the characteristic E^1_{2g} and A_{1g} Raman modes that result from a given perturbation can be expressed as:[12]

$$\Delta\omega_{E'} = -2\gamma_{E'}\omega^0_{E'}\varepsilon + k_{E'}n \quad \text{(S3)}$$

$$\Delta\omega_{A'} = -2\gamma_{A'}\omega_{A'}^{0}\varepsilon + k_{A'}n \tag{S4}$$

From this linear set of equations, we obtain relations for the strain and doping:

$$\varepsilon = \frac{k_{A'}\Delta\omega_{E'} - k_{E'}\Delta\omega_{A'}}{2\gamma_{A'}\omega_{A'}^{0}k_{E'} - 2\gamma_{E'}\omega_{E'}^{0}k_{A'}} \tag{S5}$$

$$n = \frac{\gamma_{A'}\omega_{A'}^{0}\Delta\omega_{E'} - \gamma_{E'}\omega_{E'}^{0}\Delta\omega_{A'}}{\gamma_{A'}\omega_{A'}^{0}k_{E'} - \gamma_{E'}\omega_{E'}^{0}k_{A'}} \tag{S6}$$

To determine the equation describing the strain isoline at zero doping, we set $n = 0$ and solve the system of Equations S1 and S2. Similarly, we can obtain the analogous equation for the strain-free doping line in the case of $\varepsilon = 0$.

$$\omega_{A'} = \omega_{A'}^{0} + \frac{\gamma_{A'}\omega_{A'}^{0}}{\gamma_{E'}\omega_{E'}^{0}}\left(\omega_{E'} - \omega_{E'}^{0}\right) \tag{S7}$$

$$\omega_{A'} = \omega_{A'}^{0} + \frac{k_{A'}}{k_{E'}}\left(\omega_{E'} - \omega_{E'}^{0}\right) \tag{S8}$$

Hence, we can generate a ε-n grid of strain and doping isolines, with respective gradients of $\frac{\gamma_{A'}\omega_{A'}^{0}}{\gamma_{E'}\omega_{E'}^{0}} = 0.19$ and $\frac{k_{A'}}{k_{E'}} = 6.73$, as shown in Figure S10. The dashed black lines represent the strain isolines and correspond to $\Delta\varepsilon = \pm 0.1$ % variations in the strain. A $\Delta\varepsilon > 0$ is indicative of tensile strain, whereas a negative $\Delta\varepsilon$ signifies compressive strain. Red dashes outline the doping isolines and indicate relative changes in the electron concentration of $\Delta n = \pm 0.1 \times 10^{13}$ cm^{-2}, where n-type doping is represented by $\Delta n > 0$ and p-type doping is implied by $\Delta n < 0$.

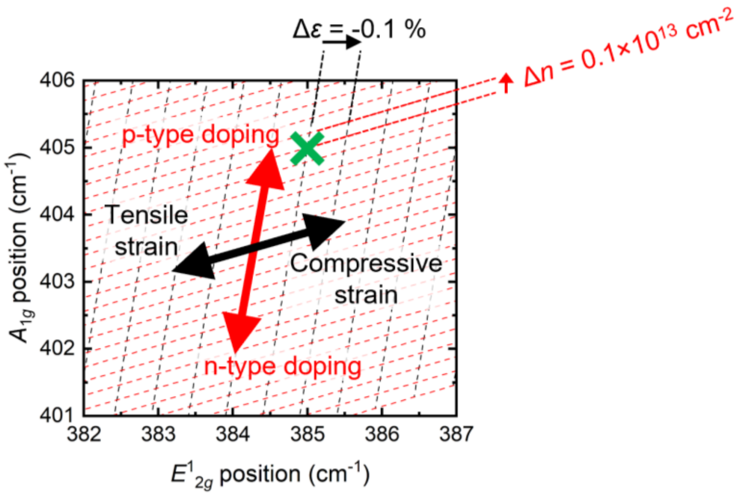

Figure S10. Representative correlative Raman plot of the A_{1g} Raman peak position as a function of the E^1_{2g} Raman peak position, with overlayed ε-n grid of isolines. The directions of the strain and doping effects indicated by the respective isolines are highlighted. The green cross indicates the intersection of the strain-free and undoped isolines at literature values of the Raman peak positions for a suspended CVD-synthesized 1L MoS_2 (E^1_{2g} ~385 cm^{-1} and A_{1g} ~405 cm^{-1}). Changes in strain and electron density relative to this point are indicated.

9. Additional XPS data for untreated, O_2 plasma-treated, and O_3-treated 1L MoS_2

Figure S11 presents high-resolution XPS scans of the O 1s core level from the untreated, oxygen (O_2) plasma exposed, and O_3-exposed 1L MoS_2 samples.

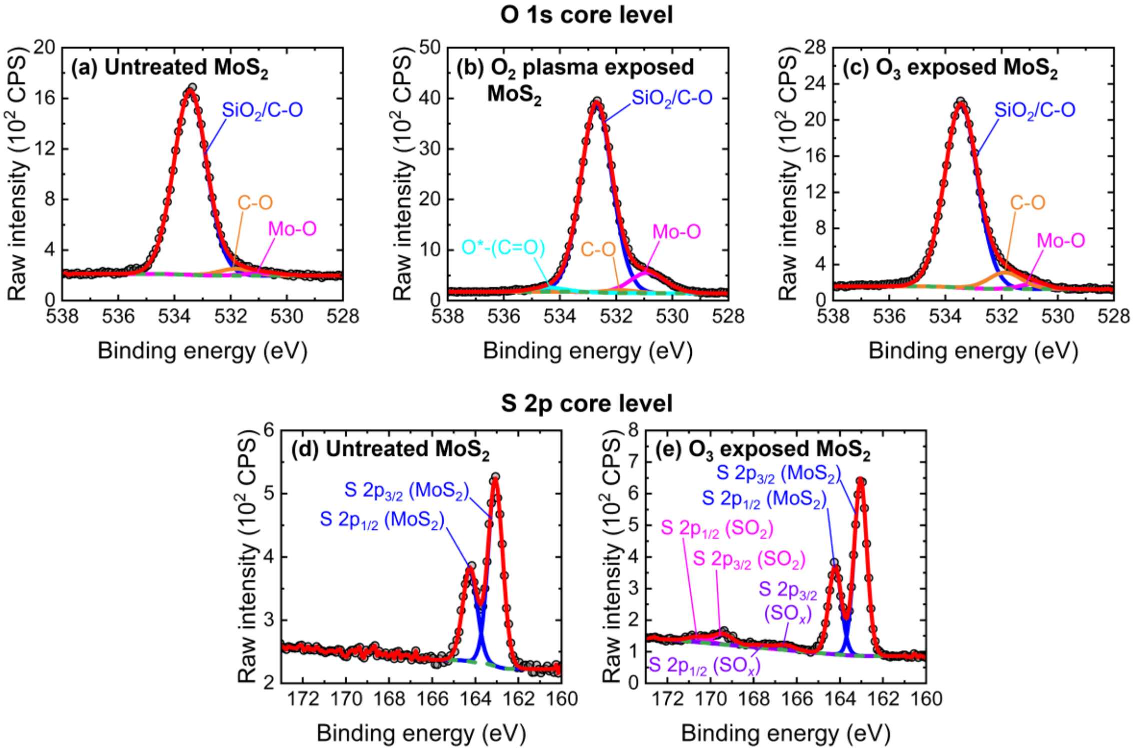

Figure S11. High-resolution, deconvoluted XPS spectra of the O 1s core level region from the (a) untreated, (b) O_2 plasma exposed, and (c) O_3-exposed 1L MoS_2 samples. High-resolution, deconvoluted XPS spectra of the S 2p core level region from the (d) untreated and (e) O_3-exposed 1L MoS_2 samples. The raw intensities are plotted in counts per second (CPS). The black circular markers represented the raw, recorded data and the solid, coloured lines correspond to fitted data. All peaks in this figure were fitted to Shirley backgrounds, with the exception of (e), where a Tougaard background was used in order to prevent the background rising above the experimental data. The background in each spectrum is indicated by the dashed green line.

References

1. Radisavljevic, B.; Radenovic, A.; Brivio, J.; Giacometti, V.; Kis, A., Single-layer MoS_2 transistors. *Nat. Nanotechnol.* **2011,** *6* (3), 147-150.
2. Splendiani, A.; Sun, L.; Zhang, Y.; Li, T.; Kim, J.; Chim, C.-Y.; Galli, G.; Wang, F., Emerging Photoluminescence in Monolayer MoS_2. *Nano Lett.* **2010,** *10* (4), 1271-1275.
3. Jeon, J.; Jang, S. K.; Jeon, S. M.; Yoo, G.; Jang, Y. H.; Park, J.-H.; Lee, S., Layer-controlled CVD growth of large-area two-dimensional MoS_2 films. *Nanoscale* **2015,** *7* (5), 1688-1695.
4. Zhang, X.; Qiao, X.-F.; Shi, W.; Wu, J.-B.; Jiang, D.-S.; Tan, P.-H., Phonon and Raman scattering of two-dimensional transition metal dichalcogenides from monolayer, multilayer to bulk material. *Chem. Soc. Rev.* **2015,** *44* (9), 2757-2785.
5. Li, H.; Zhang, Q.; Yap, C. C. R.; Tay, B. K.; Edwin, T. H. T.; Olivier, A.; Baillargeat, D., From Bulk to Monolayer MoS_2: Evolution of Raman Scattering. *Adv. Funct. Mater.* **2012,** *22* (7), 1385-1390.
6. Gołasa, K.; Grzeszczyk, M.; Bożek, R.; Leszczyński, P.; Wysmołek, A.; Potemski, M.; Babiński, A., Resonant Raman scattering in MoS_2 - From bulk to monolayer. *Solid State Commun.* **2014,** *197*, 53-56.
7. Mohiuddin, T. M. G.; Lombardo, A.; Nair, R. R.; Bonetti, A.; Savini, G.; Jalil, R.; Bonini, N.; Basko, D. M.; Galiotis, C.; Marzari, N.; Novoselov, K. S.; Geim, A. K.; Ferrari, A. C., Uniaxial strain in graphene by Raman spectroscopy: G peak splitting, Gruneisen parameters, and sample orientation. *Phys. Rev. B* **2009,** *79* (20), 205433.
8. Lee, J. E.; Ahn, G.; Shim, J.; Lee, Y. S.; Ryu, S., Optical separation of mechanical strain from charge doping in graphene. *Nat. Commun.* **2012,** *3* (1), 1024.
9. Androulidakis, C.; Tsoukleri, G.; Koutroumanis, N.; Gkikas, G.; Pappas, P.; Parthenios, J.; Papagelis, K.; Galiotis, C., Experimentally derived axial stress–strain relations for two-dimensional materials such as monolayer graphene. *Carbon* **2015,** *81*, 322-328.
10. Armano, A.; Buscarino, G.; Cannas, M.; Gelardi, F. M.; Giannazzo, F.; Schilirò, E.; Agnello, S., Monolayer graphene doping and strain dynamics induced by thermal treatments in controlled atmosphere. *Carbon* **2018,** *127*, 270-279.
11. Schilirò, E.; Lo Nigro, R.; Panasci, S. E.; Gelardi, F. M.; Agnello, S.; Yakimova, R.; Roccaforte, F.; Giannazzo, F., Aluminum oxide nucleation in the early stages of atomic layer deposition on epitaxial graphene. *Carbon* **2020,** *169*, 172-181.
12. Michail, A.; Delikoukos, N.; Parthenios, J.; Galiotis, C.; Papagelis, K., Optical detection of strain and doping inhomogeneities in single layer MoS_2. *Appl. Phys. Lett.* **2016,** *108* (17), 173102.
13. Chae, W. H.; Cain, J. D.; Hanson, E. D.; Murthy, A. A.; Dravid, V. P., Substrate-induced strain and charge doping in CVD-grown monolayer MoS_2. *Appl. Phys. Lett.* **2017,** *111* (14), 143106.
14. Panasci, S. E.; Schilirò, E.; Greco, G.; Cannas, M.; Gelardi, F. M.; Agnello, S.; Roccaforte, F.; Giannazzo, F., Strain, Doping, and Electronic Transport of Large Area Monolayer MoS_2 Exfoliated on Gold and Transferred to an Insulating Substrate. *ACS Appl. Mater. Interfaces* **2021,** *13* (26), 31248-31259.
15. Schilirò, E.; Panasci, S. E.; Mio, A. M.; Nicotra, G.; Agnello, S.; Pecz, B.; Radnoczi, G. Z.; Deretzis, I.; La Magna, A.; Roccaforte, F.; Lo Nigro, R.; Giannazzo, F., Direct atomic layer deposition of ultra-thin Al_2O_3 and HfO_2 films on gold-supported monolayer MoS_2. *Appl. Surf. Sci.* **2023,** *630*, 157476.
16. Schilirò, E.; Nigro, R. L.; Panasci, S. E.; Agnello, S.; Cannas, M.; Gelardi, F. M.; Roccaforte, F.; Giannazzo, F., Direct Atomic Layer Deposition of Ultrathin Aluminum Oxide on Monolayer MoS_2 Exfoliated on Gold: The Role of the Substrate. *Adv. Mater. Interfaces* **2021,** *8* (21), 2101117.
17. Healy, B. F. M.; Pain, S. L.; Lloyd-Hughes, J.; Grant, N. E.; Murphy, J. D., Tunable Photoluminescence from Monolayer Molybdenum Disulfide. *Adv. Mater. Interfaces* **2024,** *11* (28), 2400305.
18. Lloyd, D.; Liu, X.; Christopher, J. W.; Cantley, L.; Wadehra, A.; Kim, B. L.; Goldberg, B. B.; Swan, A. K.; Bunch, J. S., Band Gap Engineering with Ultralarge Biaxial Strains in Suspended Monolayer MoS_2. *Nano Lett.* **2016,** *16* (9), 5836-5841.

19. Rice, C.; Young, R. J.; Zan, R.; Bangert, U.; Wolverson, D.; Georgiou, T.; Jalil, R.; Novoselov, K. S., Raman-scattering measurements and first-principles calculations of strain-induced phonon shifts in monolayer MoS_2. *Phys. Rev. B* **2013,** *87* (8), 081307.
20. Chakraborty, B.; Bera, A.; Muthu, D. V. S.; Bhowmick, S.; Waghmare, U. V.; Sood, A. K., Symmetry-dependent phonon renormalization in monolayer MoS_2. *Phys. Rev. B* **2012,** *85* (16), 161403.
